# *Trypanosoma brucei* Co-opts NK Cells to Kill Splenic B2 B Cells

**DOI:** 10.1371/journal.ppat.1005733

**Published:** 2016-07-12

**Authors:** Deborah Frenkel, Fengqiu Zhang, Patrick Guirnalda, Carole Haynes, Viki Bockstal, Magdalena Radwanska, Stefan Magez, Samuel J. Black

**Affiliations:** 1 Department of Veterinary and Animal Sciences, University of Massachusetts Amherst, Amherst, Massachusetts, United States of America; 2 Laboratory for Cellular and Molecular Immunology, Vrije Universiteit Brussel, Brussels, Belgium; 3 Ghent University Global Campus, Yeonsu-gu, Incheon, South Korea; 4 Department of Structural Biology, Vrije Universiteit Brussel, Brussels, Belgium; University of Manitoba, CANADA

## Abstract

After infection with *T*. *brucei* AnTat 1.1, C57BL/6 mice lost splenic B2 B cells and lymphoid follicles, developed poor parasite-specific antibody responses, lost weight, became anemic and died with fulminating parasitemia within 35 days. In contrast, infected C57BL/6 mice lacking the cytotoxic granule pore-forming protein perforin (*Prf1* ^*-/-*^) retained splenic B2 B cells and lymphoid follicles, developed high-titer antibody responses against many trypanosome polypeptides, rapidly suppressed parasitemia and did not develop anemia or lose weight for at least 60 days. Several lines of evidence show that *T*. *brucei* infection-induced splenic B cell depletion results from natural killer (NK) cell-mediated cytotoxicity: i) B2 B cells were depleted from the spleens of infected intact, T cell deficient (*TCR* ^*-/-*^) and FcγRIIIa deficient (CD16^-/-^) C57BL/6 mice excluding a requirement for T cells, NKT cell, or antibody-dependent cell-mediated cytotoxicity; ii) administration of NK1.1 specific IgG2a (mAb PK136) but not irrelevant IgG2a (myeloma M9144) prevented infection-induced B cell depletion consistent with a requirement for NK cells; iii) splenic NK cells but not T cells or NKT cells degranulated in infected C57BL/6 mice co-incident with B cell depletion evidenced by increased surface expression of CD107a; iv) purified NK cells from naïve C57BL/6 mice killed purified splenic B cells from *T*. *brucei* infected but not uninfected mice *in vitro* indicating acquisition of an NK cell activating phenotype by the post-infection B cells; v) adoptively transferred C57BL/6 NK cells prevented infection-induced B cell population growth in infected Prf1^-/-^ mice consistent with *in vivo* B cell killing; vi) degranulated NK cells in infected mice had altered gene and differentiation antigen expression and lost cytotoxic activity consistent with functional exhaustion, but increased in number as infection progressed indicating continued generation. We conclude that NK cells in *T*. *brucei* infected mice kill B cells, suppress humoral immunity and expedite early mortality.

## Author Summary

Trypanosomiasis-susceptible mammals that are infected with African trypanosomes develop anemia, lose weight, lose immune competence and have an increased susceptibility to secondary infections. Our earlier studies in the *T*. *b*. *brucei* AnTat 1.1 mouse model of African trypanosomiasis show that infection-induced loss of humoral immune competence correlates with depletion of splenic transitional, marginal zone and follicular B cells. Here, we show that splenic B2 B cells in the infected mice acquire an NK cell activating phenotype after trypanosome wave remission and are deleted by antibody-independent, splenic NK cell-mediated cytotoxicity, which is a perforin dependent process. In the absence of this killing process, i.e., in perforin gene knock out mice, *T*. *brucei* infection did not cause depletion of splenic B cells, or disruption of splenic architecture, or anemia, or weight loss, or early mortality and did elicit high-titer trypanosome polypeptide-specific antibody responses. The studies show that *T*. *brucei* co-opts host NK cells to degrade the antibody limb of the adaptive immune system and elicit life-threatening trypanosomiasis pathology.

## Introduction

*Trypanosoma brucei brucei*, *T*. *congolense* and *T*. *vivax* cause Animal African Trypanosomiasis, which severely constrains cattle ranching and integrated agriculture in sub-Saharan Africa [1]. In addition, two sub-species of *T*. *brucei*, namely *T*. *b*. *gambiense* and *T*. *b*. *rhodesiense*, infect humans causing human African trypanosomiasis [2], which threatens about 60 million people in sub-Saharan Africa. These tsetse-transmitted protozoan parasites inhabit the blood plasma, and in the case of *T*. *brucei* (*sensu lato*) and *T*. *vivax*, also the tissue fluids of their mammal hosts. African trypanosomes evade immune elimination by antigenic variation of their expressed variable surface glycoprotein (VSG) coat, which in *T*. *brucei* occurs in “about 0.1% of trypanosome divisions”, and results from “differential expression of a VSG gene from an archive of hundreds of silent VSG genes and pseudogenes” [3]. As a result of VSG antigenic variation, African trypanosomiasis is characterized by recurring waves of parasitemia in which dominant VSG types are cleared by VSG specific antibody and the surviving antigenic variants seed the next parasitemic wave.

Peak levels of trypanosome parasitemia in trypanosomiasis-susceptible mammals, which include humans, domestic ruminants, domestic ungulates, dogs and laboratory rodents, range from <10^6^ to >10^8^ trypanosomes per ml blood depending on host species and parasite virulence. In each parasitemic wave dominant antigenic variants are tagged for phagocytosis, and in some species antibody and complement dependent lysis by IgM and IgG antibodies that are specific for VSGs expressed by dominant antigenic variants [4–6]. However, infected trypanosomiasis-susceptible hosts typically develop only short-lived, low-titer, IgG antibody responses against VSGs and other trypanosome polypeptides [7], eventually become immunosuppressed and less able to control new trypanosome antigenic variants and secondary infections [8–10], develop anemia, cachexia and reproductive disorders [11–14], and often die. In contrast, infected trypanosomiasis-resistant mammals, e.g., African Cape buffalo, rapidly constrain trypanosome parasitemia to a level of <10^2^ trypanosomes/ml blood, do not show signs of disease [15–17], and accumulate trypanostatic/trypanocidal IgG antibodies against previously expressed VSGs in their blood [17]. This raises the possibility that preservation of immune competence in infected trypanosomiasis-susceptible hosts, particularly the sustained ability to make VSG-specific IgG antibodies and associated T helper cell responses, would increase their ability to control parasitemia and decrease trypanosomiasis pathology.

B cell clonal exhaustion has been proposed as a cause of trypanosomiasis-induced suppression of humoral immune responses [18]. This view is supported by the development of lymphopenia in cattle and sheep with animal African trypanosomiasis [11, 19, 20] and with ablation of bone marrow lymphopoiesis and depletion of splenic transitional, marginal zone (MZ) cells and follicular (Fo) B cells in mice infected with *T*. *brucei* [10, 21, 22]. The loss of these B cells, which are from the B2 B cell lineage [23, 24], severely compromises trypanosome- and vaccine-specific antibody production [10]. Splenic B cell depletion also occurs in mice infected with *T*. *congolense* [25] and *T*. *vivax* [26].

Splenic B2 B cell dynamics have not been studied in cattle although an infection-induced increase in splenic B1-like B cells has been reported [27]. Analyses of peripheral blood B cell dynamics in human patients with *T*. *b*. *gambiense* show a significant increase in T cell-independent B cells and a significant decrease in T cell-dependent B cells as a percentage of total blood lymphocytes accompanied by a significant decrease in anti-measles antibody in serum relative to age-, gender- and habitat-matched uninfected individuals, although not of a level to compromise immunity [28]. It remains possible that there is a link between the infection-induced mechanism that causes the depletion of splenic B2 B cells in trypanosome-infected mice, and those that prevent the development of trypanosome-specific IgG antibodies in infected cattle [29] and cause the proportional decrease in T cell-dependent relative to T cell-independent B cells in the peripheral blood of *T*. *b*. *gambiense* infected people.

We have previously shown that depletion of splenic B2 B cells in *T*. *brucei* infected mice results from their apoptosis [10], which occurs independently of tumor necrosis factor (TNF)-, FasL- and prostaglandin-induced death pathways [22] and is thus distinct from B cell fratricide which arises in mice infected with the South American trypanosome *T*. *cruzi* [30]. Here we dissect the cellular basis of *T*. *brucei* infection-induced B2 B cell depletion using a combination of mutant mouse strains on the C57BL/6 background that lack various defined immune functions, as well as antibody-mediated depletion of putative effector cells, effector cell phenotype analysis, fate-mapping of fluorochrome-labeled cells after adoptive transfer, and *in vitro* and *in vivo* B cell killing assays. These studies show that splenic B2 B cells in *T*. *brucei-*infected mice are killed by NK cell-mediated cytotoxicity. The killing requires perforin, a pore-forming protein, which is found in cytotoxic granules of killer cells and which together with granzymes induces target cell apoptosis [31–34]. Although cytotoxic T cells and NKT cells also mediate perforin-dependent killing we show that they are not involved in killing the B2 B cells in *T*. *brucei* infected mice. NK cells are lymphocyte-like components of the innate immune system. They monitor cell surfaces using an array of receptors and kill target cells on which ligands for NK cell activating receptors predominate over ligands for inhibitory receptors [35, 36]. We show here that the B cells in *T*. brucei-infected mice acquire an NK cell activating phenotype. This paper does not address the nature of the infection-induced NK cell activating ligand(s) on B cells, but does address the fate of the activated NK cells.

## Results

### Infection-induced depletion of splenic B2 cells in immune system gene knock out mice

Splenic B2 B cells (Table 1—mAb profiles; S1 Fig—gating profiles) were quantified in uninfected (Table 2) and infected (5 x 10^3^ *T*. *b*. *brucei* Antat 1.1 i.p (Table 3) intact C57BL/6 mice and mice lacking genes that modulate leukocyte activation and effector functions. Irrespective of the mutation, infected mice became parasitemic (about 10^6^ *T*. *brucei*/ml blood) by 4 days after infection, developed peak parasitemias of between 8x10^7^ and 2x10^8^ *T*. *brucei*/ml blood depending on mutant mouse strain on day 6 after infection and, with the exception of mice lacking the gene encoding complement factor 3 (*C3* ^*-/-*^), remitted first wave parasitemia to a level of <10^6^ *T*. *brucei/*ml blood between 6 and 7 days after infection. In infected *C3* ^*-/-*^ mice the level of *T*. *brucei* parasitemia declined in a gradual fashion between 7 and 10 days after infection to reach a trough level of <10^6^ *T*. *brucei/*ml blood indicating that C3 may facilitate immune clearance of *T*. *brucei*, as previously shown for *T*. *congolense* [37].

**Table 1:** Differentiation antigen profiles of B2 B cells, NK cells and NKT cells used in multicolor flow cytometry.

Cell Type	Differentiation Antigen Expression
Transitional B cells	B220^+^, AA4.1^+^, IgM^+^
Type 1 Transitional B cell	B220^+^, AA4.1^+^, CD23^-^, IgM^+^
Type 2 Transitional B cell	B220^+^, AA4.1^+^, CD23^+^, IgM^hi^
Type 3 Transitional B cell	B220^+^, AA4.1^+^, CD23^+^, IgM^lo^
Marginal Zone B cell	IgM^+^, AA4.1^-^, CD23^lo^, CD21^+^
Follicular B cell	IgM^+^, AA4.1^-^, CD23^hi^, CD21^+^
Natural Killer cell	CD3^-^, NK1.1^+^

**Table 2:** Splenic B2 B cells in mutant C57BL/6 mouse strains.

C57BL/6 mouse stain (n = 3)	Mean # of Cells/Spleen (+/- 1SD)		
	Transitional 2+3	MZB	FoB
Wild type	2.5 x 10^6^ (4.2 x 10^5^)	1.7 x 10^6^ (4.3 x 10^5^)	2.2 x 10^7^ (3.9 x 10^6^)
*Nu* ^*+/+*^	1.2 x 10^6^ (1.4 x 10^5^)	1.8 x 10^6^ (1.1 x 10^6^)	2.4 x 10^7^ (0.2 x 10^5^)
*TCR* ^*-/-*^	3.2 x 10^6^ (5.5 x 10^5^)	1.4 x 10^6^ (1.8 x 10^5^)	2.1 x 10^7^ (1.9 x 10^6^)
*C3* ^*-/-*^	1.8 x 10^6^ (8.0 x 10^5^)	3.5 x 10^6^ (8.0 x 10^5^)	1.4 x 10^7^ (1.8 x 10^6^)
*CD16* ^*-/-*^	3.2 x 10^6^ (4.8 x 10^5^)	3.8 x 10^5^ (0.7 x 10^5^)	1.8 x 10^7^ (5.0 x 10^6^)
*MyD88* ^*-/-*^	2.0 x 10^6^ (0.9 x 10^5^)	1.9 x 10^6^ (0.8 x 10^5^)	8.7 x 10^6^ (5.8 x 10^5^)
*TRIFF* ^*-/-*^	6.4 x 10^5^ (0.2 x 10^5^)	7.4 x 10^5^ (0.7 x 10^5^)	2.2 x 10^6^ (1.7 x 10^5^)
*INFγ* ^*-/-*^	1.6 x 10^6^ (0.5 x 10^5^)	6.8 x 10^5^ (2.2 x 10^5^)	1.1 x 10^7^ (2.5 x 10^6^)
*TNFR1* ^*-/-*^	1.4 x 10^6^ (3.1 X 10^5^)	4.3 x 10^5^ (0.9 x 10^5^)	1.4 x 10^7^ (5.8 x 10^6^)
*Gal3* ^*-/-*^	1.8 x 10^6^ (2.1 x 10^5^)	8.7 x 10^5^ (2.1 x 10^5^)	1.5 x 10^7^ (1.9 x 10^6^)
*Prf1* ^*-/-*^	4.0 x 10^6^ (5.6 x 10^5^)	6.1 x 10^5^ (1.5 x 10^5^)	2.2 x 10^7^ (2.2 x 10^6^)

**Table 3:** Impact of *T*. *brucei* infection on splenic B2 B cells.

C57BL/6 mouse stain* (n = 3)	Fold change Log_2_ in splenic B2 B cells 10 days after infection of mice with *T*. *b*. *brucei* AnTat 1.1 relative to uninfected mice of the same strain (P <0.05 = significant)		
	Transitional 2+3	MZB	FoB
Wild type	-2.4 (P = 0.026)	-3.4 (p = 0.033)	-1.9 (p = 0.023)
*Nu* ^*+/+*^	-3.2 (p = 0.02)	-3.5 (p = 0.02)	-2.8 (p = 0.01)
*TCR* ^*-/-*^	-3.3 (p = 0.0001)	-2.5(p = 0.0001)	-2.8 (p = 0.0001)
*C3* ^*-/-*^	-1.9 (p = 0.24)	-3.4 (p = 0.01)	-3.3 (p = 0.0001)
*CD16* ^*-/-*^	-3.1 (p = 0.003)	-1.0 (p = 0.05)	-2.5 (p = 0.04)
*MyD88* ^*-/-*^	-1.4 (p = 0.01)	-1.6 (p = 0.013)	-1.2 (p = 0.005)
*TRIFF* ^*-/-*^	-1.5 (p = 0.006)	-1.0 (p = 0.02)	-2.0 (p = 0.004)
*INFg* ^*-/-*^	-3.0 (p = 0.04)	-1.4 (p = 0.004)	-1.1 (p = 0.03)
*TNFR1* ^*-/-*^	-3.0 (p = 0.05)	-3.3 (p = 0.004)	-1.6 (p = 0.03)
*Gal3* ^*-/-*^	-2.4 (p = 0.002)	-1.8 (p = 0.009)	-1.6 (p = 0.005)
*Prf1* ^*-/-*^	0.7 (p = 0.02)	0.4 (p = 0.43)	0 (p = 0.16)

* Results are representative of at least 2 experiments per mutant stain

**Fig 1:**
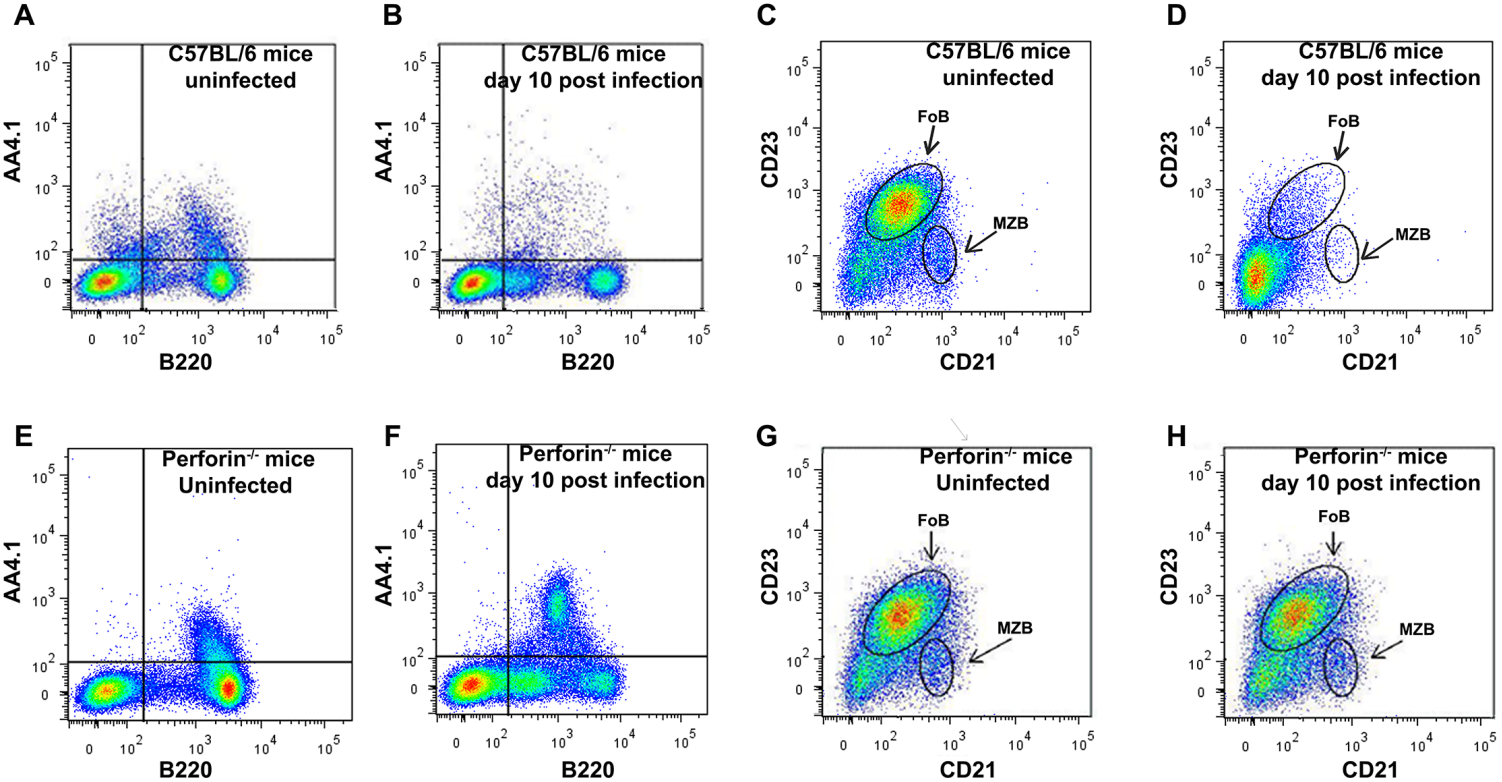
FACS analysis of splenic B2 B cells in uninfected and *T*. *brucei* ANTat 1.1-infected C57BL/6 and *Prf1* ^*-/-*^ C57BL/6 mice. **(A)** Transitional B cells (IgM^+^AA4.1^+^B220^+^) in a representative uninfected C57BL/6 mouse, **(B)** Transitional B cells in representative day 10 *T*. *brucei* infected C57BL/6 mouse; (**C)**–Marginal zone B cells (IgM^+^CD21^+^CD23^lo^) and follicular B cells (IgM+CD21+CD23^hi^) in a representative uninfected C57BL/6 mouse, **(D)** Marginal zone B cells and follicular B cells in a representative day 10 *T*. *brucei* infected C57BL/6 mice; **(E)**—Transitional B cells in a representative uninfected *Prf1* ^*-/-*^ C57BL/6 mouse, **(F)** Transitional B cells in a representative day 10 *T*. *brucei* infected *Prf1* ^*-/-*^ C57BL/6 mouse; **G** Marginal zone B cells and follicular B cells in a representative uninfected *Prf1* ^*-/-*^ C57BL/6 mouse, (H) Marginal zone B cells and follicular B cells in a representative day 10 *T*. *brucei* infected *Prf1* ^*-/-*^ C57BL/6 mice.

Because numbers of splenic transitional, marginal zone and follicular B cells varied among mutant C57BL/6 mice (Table 2), post infection changes in spleen cells are presented as the log_2_ fold change relative to uninfected mice of the same strain (Table 3). By 10 days after infection, splenic transitional, MZ and Fo B cells (B2 B cells) were severely depleted from C57BL/6 mice that: (i) were unmodified (Table 3, line 1; representative Facs plot—Fig 1A–1D) consistent with earlier studies [10, 22], (ii) were homozygous for Nu gene expression (*nu* ^*+/+*^) (Table 3, line 2) and thus athymic and largely deficient in T cells; (iii) lacked genes encoding the β and δ chains of the T cell antigen specific receptor (Table 3, line 3) and thus lacked all αβ and γδ T cells and NKT cells; (iv) lacked the gene encoding complement factor 3 (C3, Table 3, line 4) and were thus unable to mediate antibody- and complement-dependent cell lysis, and C3b- and iC3b-dependent opsonization, (v) lacked the gene encoding CD16 (Table 3, line 5) and thus lacked the low-affinity Fcγ receptor which is required for antibody-dependent cell-mediated cytotoxicity; (vi) lacked genes encoding Toll like receptor adaptor proteins MyD88 (Table 3, line 6) and TRIFF (Table 3 line 7) and thus had defective TLR signaling, (vii) had a disrupted gene encoding interferon gamma (INFγ) and made little or no interferon gamma [38] (Table 3, line 8; spleen cell numbers and representative Facs plots—S2 Fig), (viii) lacked the gene encoding TNFR1 (Table 3, line 9) and thus lacked the lymphotoxin α-induced death pathway, and (ix) lacked the gene encoding galectin 3 (Table 3, line 10), which is implicated in trypanosomiasis-associated inflammation and anemia [39]. In contrast, by 10 days after infection of *Prf1* ^*-/-*^ mice with *T*. *b*. *brucei* Antat 1.1, splenic transitional and marginal zone B cells increased slightly while numbers of follicular B cells remained similar to those in uninfected mice (table 3, line 11; representative Facs plots Fig 1E–1H). Thus, of the immune components studied, only perforin, which is encoded by *Prf1*, had a non-redundant function required for the early (day 10) depletion of transitional, MZ and Fo B cells from the spleens of *T*. *brucei* infected mice.

### Pathology in intact and *Prf1* ^*-/-*^ C57BL/6 mice infected with *T*. *b*. *brucei* AnTat 1.1

#### Parasitemia, weight loss, anemia

**Fig 2:**
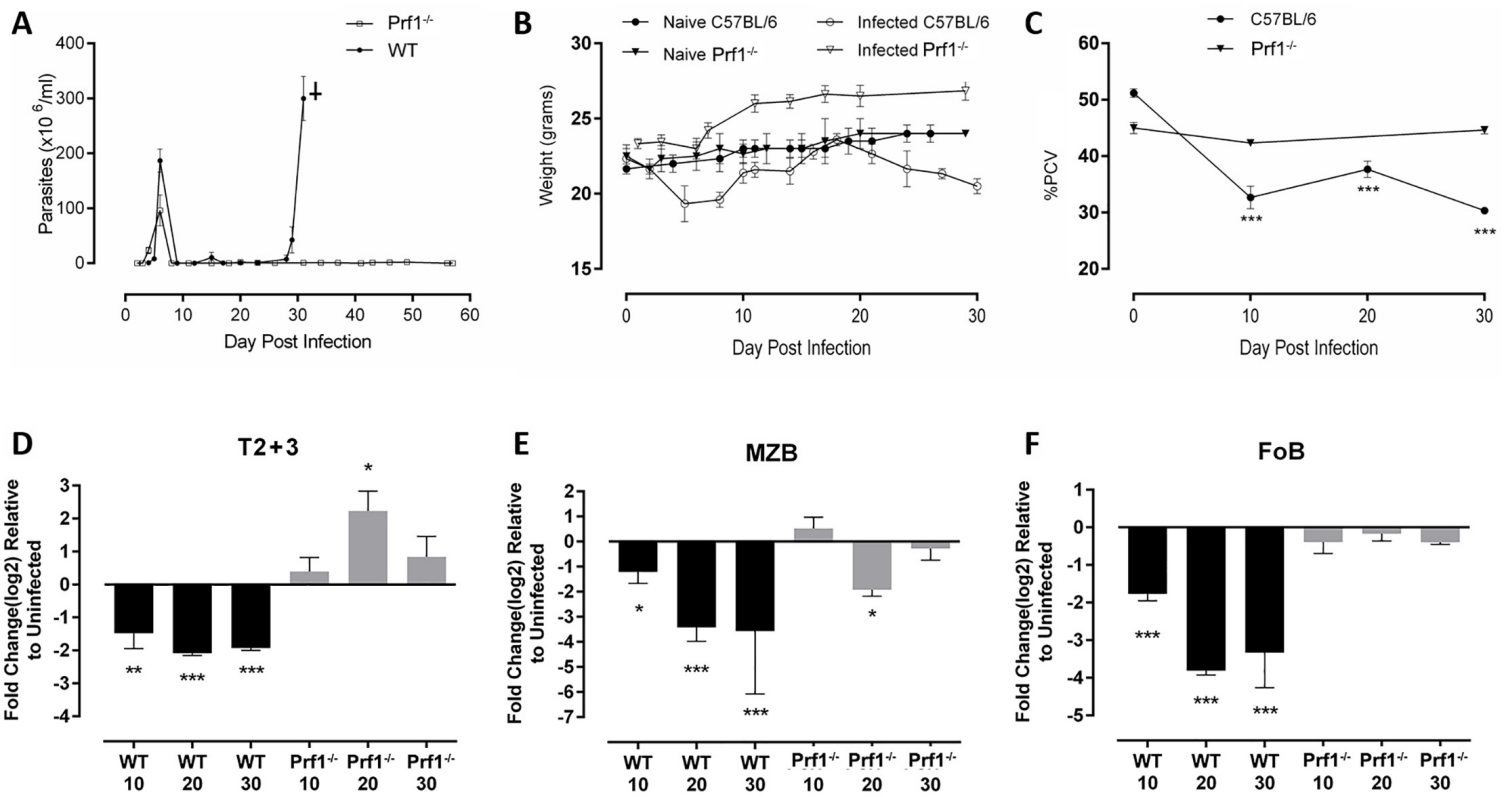
Splenic B cells and parameters of infection in intact and *Prf1* ^*-/-*^ C57BL/6 mice infected for up to 30 days with *T*. *brucei* AnTat 1.1. Parasitemia, body weight and blood packed cell volume were monitored in uninfected C57BL/6 and *Prf1* ^*-/-*^ mice, and mice infected for 10, 20 or 30 days with 5X10^3^ exponentially growing *T*. *brucei* AnTat 1.1 (n = 3/group/infection time point). In addition, spleen cells were harvested, stained with mAb to define transitional and mature B2 B cells as described in Table 1 (n = 3/group/post-infection time point, with uninfected controls processed together with infected mice at each time point) and analyzed using flow cytometry using gates shown in S2 Fig. Results are presented as: **(A)** *T*. *brucei* x 10^6^/ml blood (open squares = *Prf1* ^-/-^ C57BL/6, closed circles = C57BL/6), **(B)** body weight in grams (closed circles = uninfected C57BL/6, open circles = infected C57BL/6, closed triangles = uninfected *Prf1* ^*-/-*^ mice, open triangles = infected *Prf1* ^*-/-*^ mice), **(C)** Blood packed cell volume (PCV) (closed circles = C57BL/6 mice, closed triangles = *Prf1* ^*-/-*^ mice) measured as the % of blood cell pellet to total volume, **(D)** total numbers of transitional (types T2+T3) B cells/ spleen, **(E)** total numbers of Marginal Zone B cells/spleen, **(F)** total numbers of Follicular B cells/spleen. Data are presented as mean +/- 1 standard deviation. Significance (*<0.05, **<0.001, ***<0.0001) was determined using one-way ANOVA and Tukey’s HSD test comparing uninfected controls to infected individuals within a mouse strain but not between strains. Results are representative of 3 identical experiments.

C57BL/6 and *Prf1* ^*-/-*^ C57BL/6 mice were infected with 5 x 10^3^ *T*. *brucei* AnTat 1.1 ip at the same time, or sham infected by injection of PBS, and mice were analyzed at intervals thereafter for parasitemia, weight, blood packed cell volume, number of cells in the spleen, numbers of transitional, marginal zone and follicular B cells in the spleen, serum trypanosome specific antibody levels and spleen structure. Both strains of infected mice remitted first wave parasitemia between 6 and 7 days after infection. The peak level of 1^st^ wave parasitemia was lower in infected *Prf1* ^*-/-*^ (Fig 2A, mean 10^8^ *T*. *brucei*/ml blood) compared to infected intact mice (Fig 2A, mean 1.9 x 10^8^ *T*. *brucei*/ml blood). Thereafter, parasitemia was periodically detected (between 10^6^ and 5x10^6^ *T*. *brucei*/ml blood) for 60 days in the *Prf1* ^*-/-*^ mice when the study was stopped (Fig 2A). In contrast, infected intact mice lost control of trypanosome parasitemia by 30 days after infection and died with fulminating parasitemia by 35 days after infection (Fig 2A). Loss of control of parasitemia in infected intact mice followed a period of weight loss and anemia, which did not occur in the infected *Prf1* ^*-/-*^ mice (Fig 2B and 2C). Infected *Prf1* ^*-/-*^ mice, for the most part, maintained populations of splenic Transitional Type B cells, MZB cells and FoB cells at pre-infection levels throughout infection whereas these cell populations were substantially depleted from the spleens of infected intact mice by day 10 after infection and remained so thereafter (Fig 2D–2F).

#### Trypanosome-specific antibody responses

**Fig 3:**
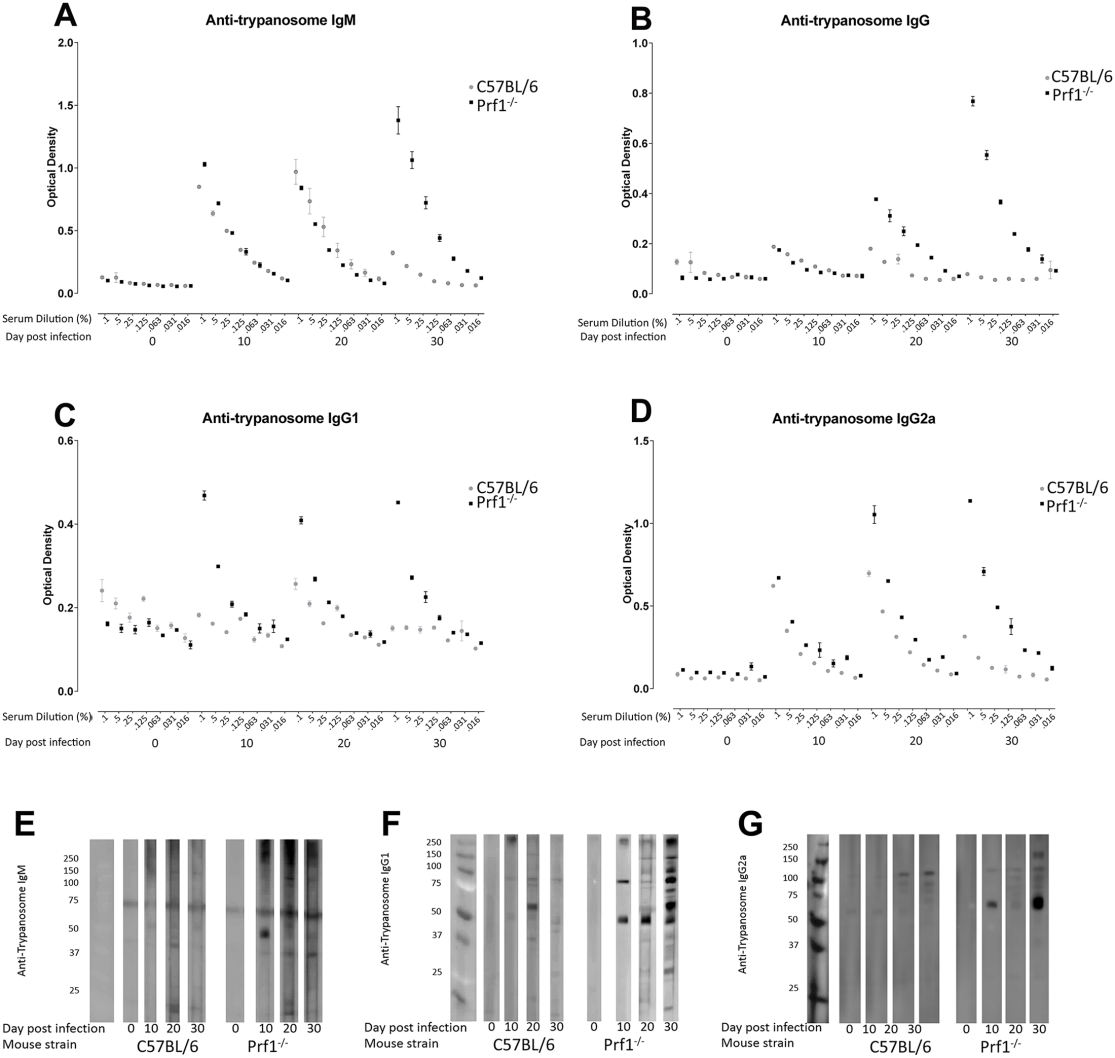
Analysis by ELISA and western blotting of trypanosome antigen specific antibodies in sera from intact and *Prf1* ^*-/-*^ C57BL/6 mice infected for up to 30 days with *T*. *brucei* AnTat 1.1. *T*. *b*. *brucei* AnTat 1.1 lysate (1 μg protein in 0.05% Np40/well) was coated on wells of an ELISA plate and used to detect trypanosome antigen specific antibodies of: (**A**) IgM, **(B)** IgG, **(C)** IgG1, **(D)** IgG2a classes in dilutions of serum (doubling dilutions from 1:1000 to 1:54,000) from groups of uninfected (naïve) intact C57BL/6 (gray dots) and *Prf1* ^*-/-*^ C57BL/6 mice (black squares) and mice that had been infected for 10, 20 or 30 days with *T*. *brucei* AnTat 1.1(n = 6/group). Trypanosome lysate (0.5% NP40) was subjected to SDS-PAGE, transferred to membrane and stained with pooled serum from a group of uninfected intact or *Prf1* ^*-/-*^ mice (n = 6), or mice infected with *T*. *brucei* AnTat 1.1 for 10, 20 or 30 days as indicated (n = 6/group) to detect polypeptide-specific antibodies of: **(E)** IgM, **(F)** IgG1 and **(G)** IgG2a classes. Results are representative of results obtained in individual mice of each group in 3 identical experiments.

Infected *Prf1* ^*-/-*^ mice mounted higher-titer IgM (Fig 3A), IgG (Fig 3B), IgG1 (Fig 3C) and IgG2a (Fig 3D) antibody responses against *T*. *brucei* AnTat 1.1 components present in 0.5% NP40 lysate than infected intact mice during the first 30 days after infection, as shown by ELISA. Furthermore the infected *Prf1* ^*-/-*^ mice produced antibodies, particularly IgG1 antibodies, against a wider range of trypanosome polypeptides in the trypanosome lysate than similarly infected intact mice, determined by Western blotting after SDS-PAGE (Fig 3E–3G).

#### Spleen structure

**Fig 4:**
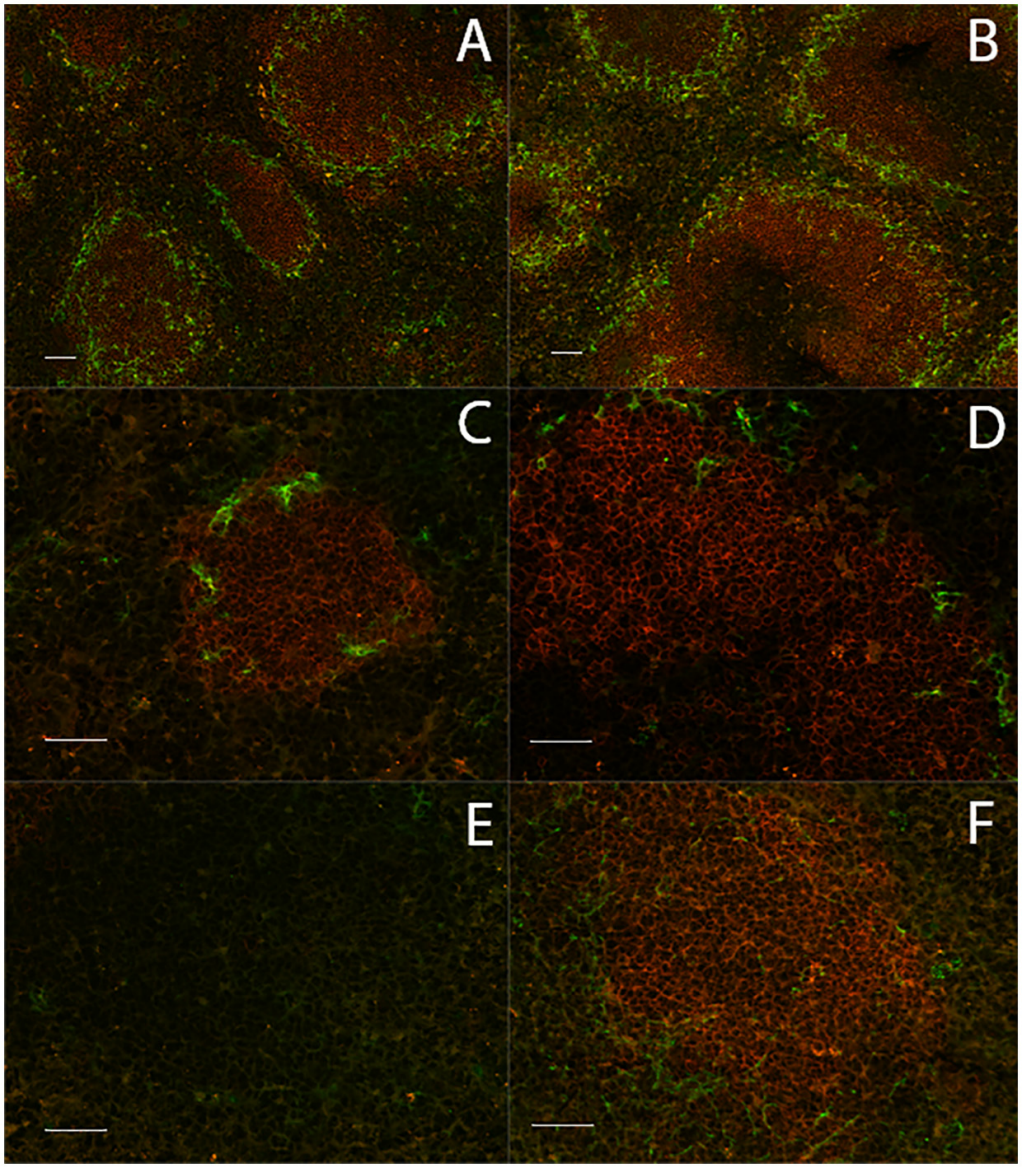
Distribution of B220^+^ and MOMA^+^ cells in spleens of intact and *Prf1* ^*-/-*^ mice infected with *T*. *brucei* AnTat 1.1. Thin sections (10 μm) of OCT embedded frozen mouse spleens, were air dried, fixed in acetone, rehydrated and stained with anti-B220 (red) to detect B cells and anti-MOMA (green) to detect marginal metallophilic macrophages. The slides were stained and read on the same day on a Zeiss MOT200 inverted microscope with a Zeiss apotome at 20x magnification. Scale bars, 50 micrometers. (**A)** Uninfected C57BL/6 mice, (**B)** uninfected *Prf1* ^*-/-*^ C57BL/6 mice, (**C, E)** C57BL/6 mice infected for 10 and 30 days with *T*. *brucei* AnTat 1.1, **(D, F)** *Prf1* ^*-/-*^ C57BL/6 mice infected for 10 and 30 days with *T*. *brucei* AnTat 1.1. Results are representative of 5 mice studied in each group.

B cell follicles, identified in cross sections of spleen as a large cluster of B220^+^ cells with a rim of MOMA^+^ marginal zone macrophages, were numerous and similar in distribution in uninfected C57BL/6 mice and *Prf1* ^*-/-*^ C57BL/6 mice (Fig 4A and 4B). However, the follicles were for the most part lost from the spleens of infected intact mice by 10 days post infection (dpi) [10] and those few follicles that remained were smaller than follicles in uninfected mice and had a substantially decreased presence of MOMA^+^ cells (Fig 4C). B cell follicles were completely absent from the spleens of C57BL/6 mice at 30 dpi (Fig 4E), but remained prevalent in the spleens of *Prf1* ^*-/-*^ C57BL/6 mice. Despite retaining large numbers of splenic lymphoid follicles, there was a decreased presence and staining intensity of MOMA^+^ cells in infected *Prf1* ^*-/-*^ mice (Fig 4D and 4F), which requires further investigation.

#### Survival

*T*. *brucei* Antat 1.1 infected *Prf1* ^*-/-*^ mice lived at least twice as long as similarly infected intact C57BL/6 mice. Despite their effective control of parasitemia, about 80% of infected *Prf1* ^*-/-*^ mice developed ruffled fur by 70 days after infection. Autopsy of these animals at 75 days post-infection showed that they had normal levels of blood packed cell volume, body weight and splenic B2 B cells relative to matched control mice, but had multiple hemorrhages of unknown etiology along the small intestine. *Trypanosoma brucei* AnTat 1.1-infected *Prf1* ^*-/-*^ mice that did not develop ruffled fur were subjected to autopsy at 90 days post infection. These mice did not have hemorrhaging of the alimentary canal or other organs, their blood packed cell volume and splenic B2 B cell content were similar to those of age-matched uninfected mice, and trypanosomes were not detected by microscopic examination of blood or spleen cell suspensions although this does not preclude sustained cryptic infection.

### Contribution of T cells, NKT cells and NK cells to *T*. *b*. *brucei* AnTat 1.1 infection-induced B cell deletion

**Fig 5:**
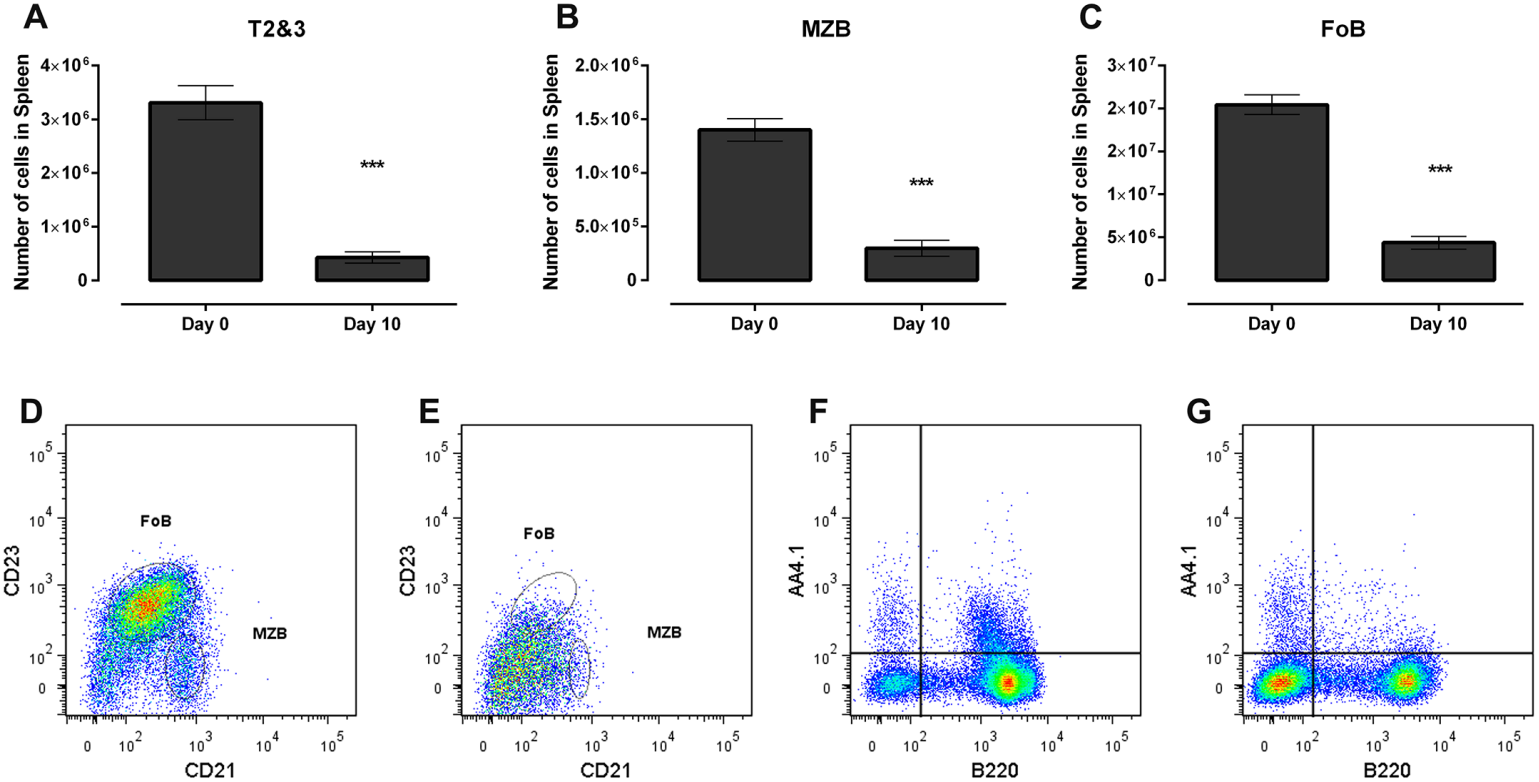
Splenic B cell populations in uninfected and *T*. *brucei* AnTat 1.1-infected TCR-/- C57BL/6 mice. Splenic B cells in uninfected B6-TCR^-/-^ mice (n = 3) and B6-TCR^-/-^ (n = 3) mice that had been infected with *T*. *brucei* AnTat 1.1 for 10 days were stained for surface markers used to define transitional and mature B2 B cells as described in Table 1 and analyzed using flow cytometry. Results are presented as individual and mean +/- 1SD numbers of: **(A)** Transitional 2+3 B cells/spleen, **(B)** MZB cells/spleen and **(C)** FoB cells/spleen. Significance (*p<0.05; **p<0.01) is determined using one-way ANOVA and Tukey’s HSD test comparing uninfected controls to infected individuals. Results are representative of 3 identical experiments. **D–G** Representative FACS plots of: **(D)** FoB and MZB spleen cells from an uninfected TCR^-/-^ C57BL/6 mouse, **(E)** FoB and MZB spleen cells from a day 10 infected TCR^-/-^ C57BL/6 mouse, **(F)** Transitional (AA4.1+, B220+) **s**pleen cells from an uninfected TCR^-/-^ C57BL/6, **(G)** Transitional (AA4.1+, B220+) **s**pleen cells from an a day 10 infected TCR^-/-^ C57BL/6 mouse.

Perforin is found in cytotoxic granules of cytotoxic T cells (CTL), NKT cells and NK cells, any or all of which might therefore mediate depletion of splenic B2 B cells in *T*. *brucei* infected mice. However numbers of transitional, marginal zone and follicular B cells were significantly decreased in *TCR* ^*-/-*^ mice infected for 10 days with *T*. *b*. *brucei* AnTat 1.1 compared to uninfected *TCR* ^*-/-*^ mice (Table 3 line 3, Fig 5A–5G), showing that splenic B2 B cell depletion did not require TCR^+^ cells, ie., T cells or NKT cells.

#### mAb PK136 NK1.1 depletion

**Fig 6:**
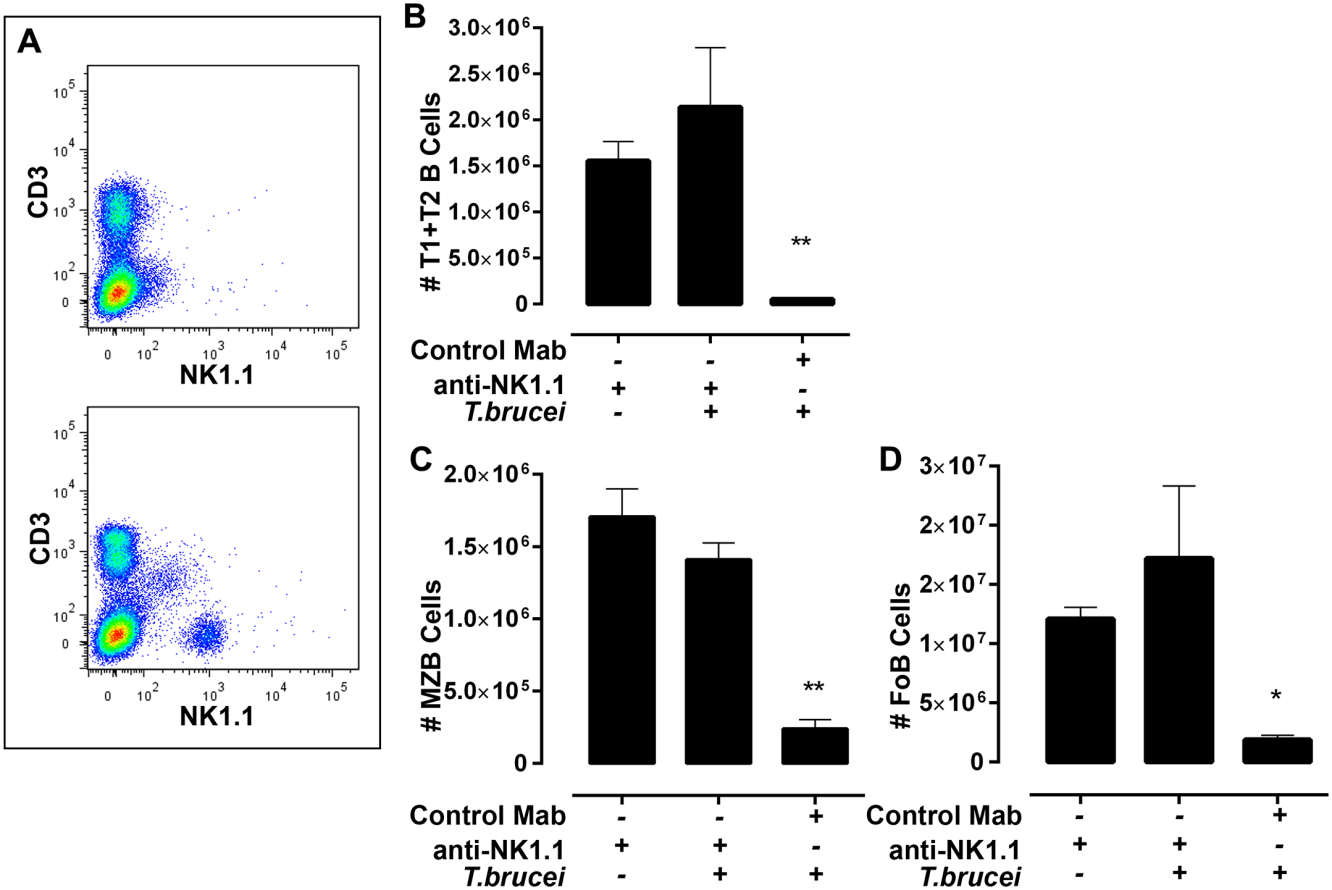
Efficacy of NK1.1 cell depletion and its impact on splenic B2 B cell survival in C57BL/6 mice infected with *T*. *brucei* AnTat 1.1. **(A)** NK1.1^+^ CD3^+^ (NKT) and NK1.1^+^ CD3^-^ (NK) cells in spleens of C57BL/6 mice (n = 3) administered 500ug mAb PK136 anti-NK1.1 monoclonal antibody (upper panel) or C57BL/6 mice (n = 3) administered an irrelevant IgG2a monoclonal antibody (lower panel; MP144 Sigma) ip on days 0, 3, and 7 and collected on day 10, **(B-D)** mean +/- 1 SD and individual total numbers of: **(B)** splenic T1 and T2 transitional type B cells, **(C)** marginal zone B cells and **(D)** follicular B cells in uninfected or *T*. *brucei* AnTat 1.1 infected C57BL/6 mice treated with anti-NK1.1 or control mAb for 10 days. Significance (*<0.05) was determined using one-way ANOVA and Tukey’s HSD test comparing uninfected controls to infected individuals. Results are representative of 4 identical experiments.

To determine whether NK cells were required for *T*. *brucei* infection-induced B2 B cell depletion, these cells were eliminated from infected mice by administration of the NK1.1 specific monoclonal antibody PK136. A single injection ip of 500 μg monoclonal anti-NK1.1 IgG2a (mAb PK136 [40–43]) but not 500 μg of an irrelevant IgG2a mouse myeloma protein (M9144), resulted in the almost complete loss of NK1.1^+^ spleen cells from C57BL/6 mice within 4 days and subsequent mAb PK136 administration on days 4, and 7 prolonged their depletion for 10 days (Fig 6 panel A). Splenic B2 B cells, which do not express NK1.1, were not affected by mAb PK136 treatment (Fig 6 panels B-D, histogram 1) or by treatment with the irrelevant IgG2a myeloma protein M9144. Administration of anti-NK1.1 mAb 136 but not irrelevant IgG2a myeloma protein to C57BL/6 mice 1 day prior to and on days 3 and 7 after infection with *T*. *b*. *brucei* AnTat 1.1 prevented infection-induced depletion of splenic B cells (Fig 6B–6D).

Although splenic B2 B cells are depleted from infected *TCR* ^*-/-*^ C57BL/6 mice (Fig 5), *TCR* ^*-/-*^ mice that were administered PK136 mAb on days 0, 3 and 7 post infection with *T*. *brucei* AnTat 1.1 retained similar numbers of splenic B2 B cells to uninfected TCR^-/-^ mice (S3 Fig; the study was repeated twice with the same result; control treatments with the irrelevant IgG2a were not done). Taken together results presented in Figs 5, 6 and S3 show that NK1.1^+^ TCR^-^ cells, i.e., NK cells are required for *T*. *brucei* infection-induced depletion of splenic transitional, MZ and Fo B cells. Depletion of splenic B2 B cells by NK cells was a result of direct NK cell-mediated cytotoxicity rather than antibody-dependent NK cell-mediated cytotoxicity because numbers of transitional, marginal zone and follicular B cells were significantly and substantially decreased in *T*. *brucei* infected *CD16* ^*-/-*^ mice compared to uninfected *CD16* ^*-/-*^ mice (Table 3 line 5, S4 Fig). *CD16* encodes FcγRIIIa, the low affinity IgG Fc region receptor, which is required for antibody-dependent cell-mediated cytotoxicity [44].

### NK cell cytotoxic granule degranulation in infected mice

**Fig 7:**
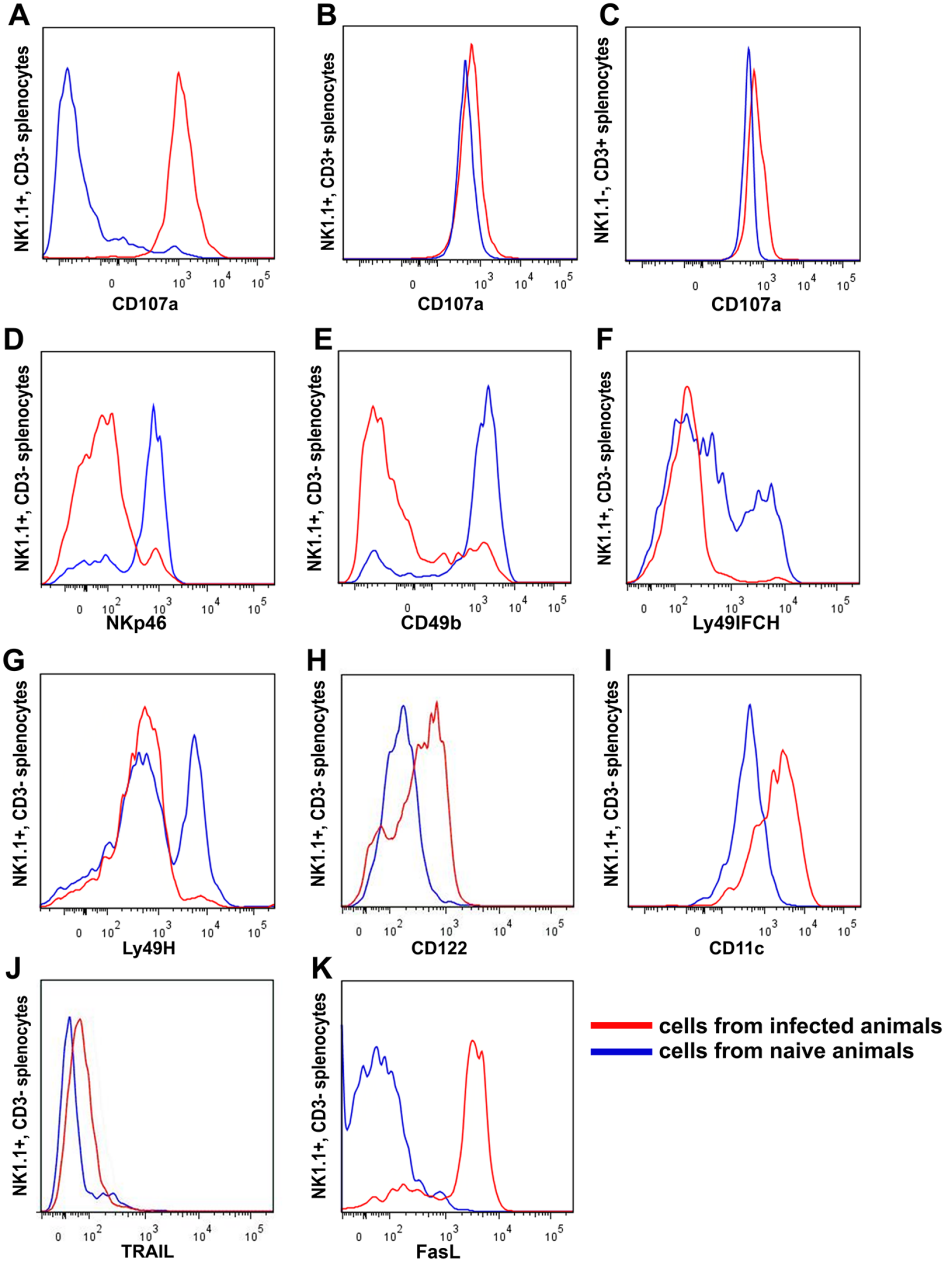
*T*. *brucei* AnTat1.1 infection-induced changes in NK cell (CD3^-^ NK1.1^+^) differentiation antigen expression. Blue lines present results obtained with NK cells from an uninfected mouse and red lines present results obtained with cells from a day 10 *T*. *brucei* AnTat 1.1 infected mouse. Results are representative of 3 mice in each experimental group and of 3 identical repeat experiments. **(A)** CD107a splenic NK cells [CD3^-^ NK1.1^+^], **(B)** CD107a splenic NKT cells [CD3^+^ NK1.1^+^], **(C)** CD107a splenic T cells [NK1.1^-^ CD3^+^] **(D)** NKp46 spleen, **(E)** CD49b spleen, **(F)** Ly49 pan (IFCH) spleen, **(G)** Ly49H spleen, **(H)** CD122 spleen, **(I)** CD11c spleen, **(J)** TRAIL spleen, **(K)** FasL spleen.

To determine whether NK cells in *T*. *brucei* infected mice express their cytolytic function, these cells were examined for surface expression of CD107a, which is a marker of secretory lysosomes including cytotoxic granules. CD107a is required for delivery of perforin to NK cell cytotoxic granules from trans-Golgi transport vesicles, and is exported onto the surface of cytotoxic cells when they degranulate during execution of their cytotoxic function [45–49]. The portion of splenic NK cells expressing CD107a on their surface increased from <5% in uninfected mice to almost 100% at 10 days after infection with *T*. *b*. *brucei* AnTat 1.1 (day 10 only shown in Fig 7A). This was accompanied by only a minimal increase in expression of CD107a by splenic CD8 T cells and NKT cells. Binding of the CD107a-specific antibody to T and NKT cells was constitutively high in C57BL/6 mice relative to binding to the NK cells (Fig 7B & 7C); this was not an experimental artifact because the splenic NK, NKT and T cells were stained in the same tube. CD107a expression also increased on NK cells harvested from the liver and lymph nodes after parasite wave remission (day 10 post infection data shown in S5 Fig) indicating that NK cells expressed their cytotoxic function, in multiple organs. Fig 7 also shows changes in expression of several other antigens by NK cells from infected mice, and these are discussed later in the text.

### Transfer of C57BL/6 NK cells into *Prf1* ^*-/-*^ mice

An adoptive transfer system was used to determine whether NK cells from C57BL/6 mice cause splenic B cell depletion in *T*. *brucei-*infected *Prf1* ^*-/-*^ mice. The C57BL/6 NK cells were isolated using negative selection and magnetic bead sorting (Miltenyi Corp., MACS micro beads) from cell suspensions prepared from pooled spleens of uninfected C57BL/6 mice. A total of 2 x 10^6^ viable NK cells were recovered/processed spleen. The isolated NK cells were labeled with e-Fluor 670 *in vitro* washed in physiologic phosphate buffered saline (PBS) and injected intravenously (iv) (5 x 10^6^ NK cells/recipient mouse) into uninfected *Prf1* ^*-/-*^ mice or *Prf1* ^*-/-*^ mice that had been infected 6 days earlier with *T*. *b*. *brucei* AnTat 1.1 which is 1 day prior to parasite wave remission (Fig 2). Spleens were harvested from recipient mice 48 and 96 hours later (corresponding to 8 and 10 days after infection), cell suspensions made and analyzed for content of eFluor 670 labeled NK cells and B2 B cells using multicolor flow cytometry.

Between 8% and 10% (4 to 5 x 10^5^ cells) of transferred eFluor 670-labeled NK cells that had been isolated from the spleens of uninfected mice were recovered from each recipient’s spleen 48hr after injection. A similar number of eFluor 670-labeled C57BL/6 NK cells were recovered from the spleens of uninfected *Prf1* ^*-/-*^ recipients 96 hours after transfer. However no eFluor 670-labeled C57BL/6 NK cells were recovered from the infected *Prf1* ^*-/-*^ mice 96 hours after transfer. It is unclear whether the labeled cells hat were transferred into infected recipients lost their efluor 670 label or died in, or migrated out of, the infected spleen between 48 and 96 hours after transfer. Similar adoptive studies were attempted with efluor 670 labeled NK cells that had been isolated from spleens of day 10 infected C57BL/6 mice. These were not recovered from the spleens of either uninfected or day 6 infected recipient C57BL/6 mice at 24 or 48 hours after iv injection; we did not determine whether these previously activated NK cells fail to localize to the recipients’ spleens, or localize and rapidly die.

**Fig 8:**
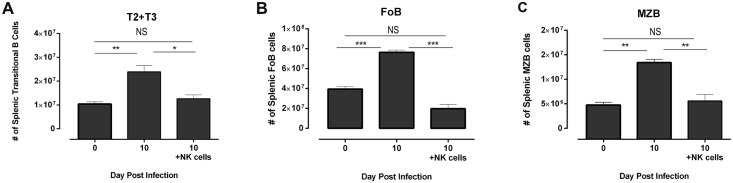
Depletion of splenic B cells in *T*. *brucei* AnTat 1.1 infected *Prf1* ^*-/-*^ mice by adoptively transferred C57BL/6 splenic NK cells. Prf1^-/-^ mice were injected ip with 5x10^3^ *T*. *brucei* Antat 1.1 (n = 6; infected) or with PBS (n = 3; uninfected). Five days later, 3 of the infected mice were injected iv., with 5x10^6^ purified C57BL/6 splenic NK cells in 0.2ml PBS/recipient, and the other 3 infected mice were injected iv with 0.2 ml PBS/recipient. Spleens were harvested from recipient mice 96 hours later (corresponding to 9 days after infection), cell suspensions prepared and **(A)** transitional Type 2 and 3 B cells, **(B)** follicular B cells, and **(C)** marginal zone B cells quantified by multicolor flow cytometry. Significance (*<0.05, **<0.01, ***<0.001) was determined using one-way ANOVA and Tukey’s HSD test comparing uninfected controls to infected individuals. Results are representative of 2 identical experiments.

The iv injection of naïve C57BL/6 NK cells into uninfected *Prf1* ^*-/-*^ recipients did not affect numbers of splenic B2 B cells up to 4 days after injection. Intravenous injection of naïve C57BL/6 NK cells into *Prf1* ^*-/-*^ mice that had been infected 6 days earlier with *T*. *brucei* had no effect on splenic B cell numbers at day 8 after infection relative to infected mice that had been injected iv with PBS. However, splenic B2 B cell numbers were significantly decreased at day 10 after infection relative to those in infected mice injected iv with PBS, but not relative to uninfected *Prf1* ^*-/-*^ mice (Fig 8). The results are consistent with modest depletion of splenic B2 B cells in infected *Prf1* ^*-/-*^ mice by the C57BL/6 NK cells.

### Direct NK cell-mediated B cell killing *in vitro*

**Fig 9:**
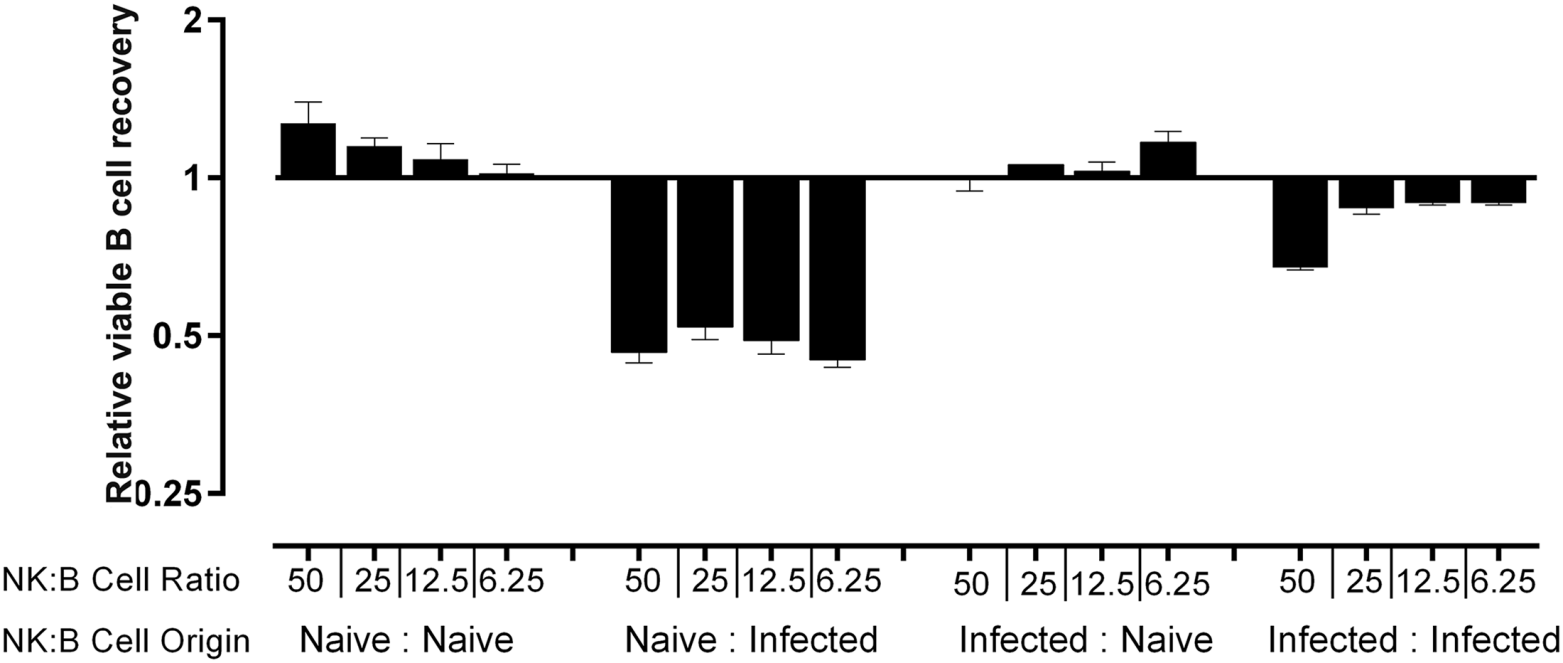
NK cells selectively kill splenic B cells from mice infected with *T*. *brucei* AnTat 1.1. eFluor 670-labeled B cells from naive mice or mice infected with *T*. *brucei* 8 days earlier (10^5^ B cells/well) were incubated at 37°C with or without NK cells from naïve mice or mice infected with *T*. *brucei* 9 days earlier to give ratios of between 50 and 0.25 NK cells/1B cell. After 3 hr incubation viable B cells were enumerated in each well. Results are presented as: relative viable B cell recovery, where 1 is the number of viable B cells remaining in cultures after incubation in the absence of NK cells. This was 8 x 10^4^ B cells from uninfected mice/well, and 5 x 10^4^ B cells from infected mice/well). The study was repeated 3 times with similar results.

To determine whether NK cells directly kill B cells from infected mice, NK cells and resting B2 B cells were isolated using negative selection and magnetic bead sorting (Miltenyi Corp., MACS micro beads) from the spleens of uninfected mice, and from mice infected with *T*. *b*. *brucei* AnTat1.1 either 8 days earlier (for post-infection B cells) or 10 days earlier (for post-infection NK cells). The B cells were labeled with eFluor 670 *in vitro*, washed in PBS and the populations mixed at NK to B cell ratios ranging from 50:1 to 6.25:1 holding the number of target cells (B cells) constant, which is a standard killing protocol [50]. B cells were also incubated in the absence of NK cells. The cell mixtures, in duplicate, were incubated *in vitro* at 37°C in a humid atmosphere of 5% CO_2_ in air for 3 hours after which the vital dye 7AAD and a fixed number of fluorescent beads was added to each culture to facilitate quantitation of viable B cells (7AAD^-^ IgM^+^ eFluor 670^+^) by flow cytometry. NK cells from uninfected or *T*. *brucei* infected mice did not kill B cells from uninfected mice even at a ratio of 50 NK cells to 1 B cell (Fig 9). However during 3 hours of incubation *in vitro* and at all NK cell to B cell ratios tested, NK cells from the uninfected mice killed >50% splenic B cells isolated from mice that had been infected 8 days earlier with *T*. *brucei* AnTat 1.1 (Fig 9). Interestingly, NK cells from the infected mice showed some B cell killing at a ratio of 50 NK cells to 1 B cell but lost killing activity at lower NK cell to B cell ratios. In contrast, NK cells from uninfected mice maximally killed B cells from infected mice even at a ratio of 6.5 NK cells/ B cell and in repeat experiments maximal killing was observed at 1 or fewer NK cells/B cell, consistent with serial killing of a magnitude similar to that shown by IL-2 activated human NK cells [51].

### *T*. *brucei* infection-induced changes in NK cell phenotype and transcriptome

Results presented in Fig 9 show that NK cells collected from the spleens of mice that had been infected with *T*. *brucei* for 10 days had much lower B cell killing activity compared to NK cells from uninfected mice consistent with exhaustion of cytolytic function by prior cytotoxic activity as denoted by increased levels of surface CD107a (Fig 7A). Day 10 post infection splenic NK cells also differed from splenic NK cells of naïve mice in several other ways. Whereas NK cells from uninfected mice expressed the natural cytotoxicity receptor NKp46 (Fig 7D blue) and the integrin alpha subunit CD49b (Fig 7E blue), expression of these differentiation antigens was greatly decreased on NK cells from day 10 infected mice (Fig 7D & 7E red). In addition, while about 40% of the NK cells from uninfected mice cells had high expression of receptors for MHC class I detected by a Ly49 I F C H pan specific mAb and for m157 MCMV protein detected by an Ly49 H specific mAb (Fig 7F & 7G blue) as previously reported [52], expression of these receptors was greatly decreased on NK cells from day 10 infected mice (Fig 7F & 7G red). In contrast, whereas few NK cells from uninfected mice expressed the common β chain of the IL-2R/IL-15R, CD122 (Fig 7H blue), and death ligands TRAIL (Fig 7J blue) and Fas L (Fig 7K blue), their expression, particularly that of FASL was increased on NK cells from day 10 infected mice (Fig 7H, 7J and 7K red). Furthermore, expression of CD11c was weak on NK cells from uninfected mice but substantially greater on NK cells from day 10 infected mice (Fig 7I), a characteristic that is shared with NK dendritic cells although dendritic NK cells also express CD49b [53] distinguishing them from the splenic NK cells of day 10 infected mice.

**Fig 10:**
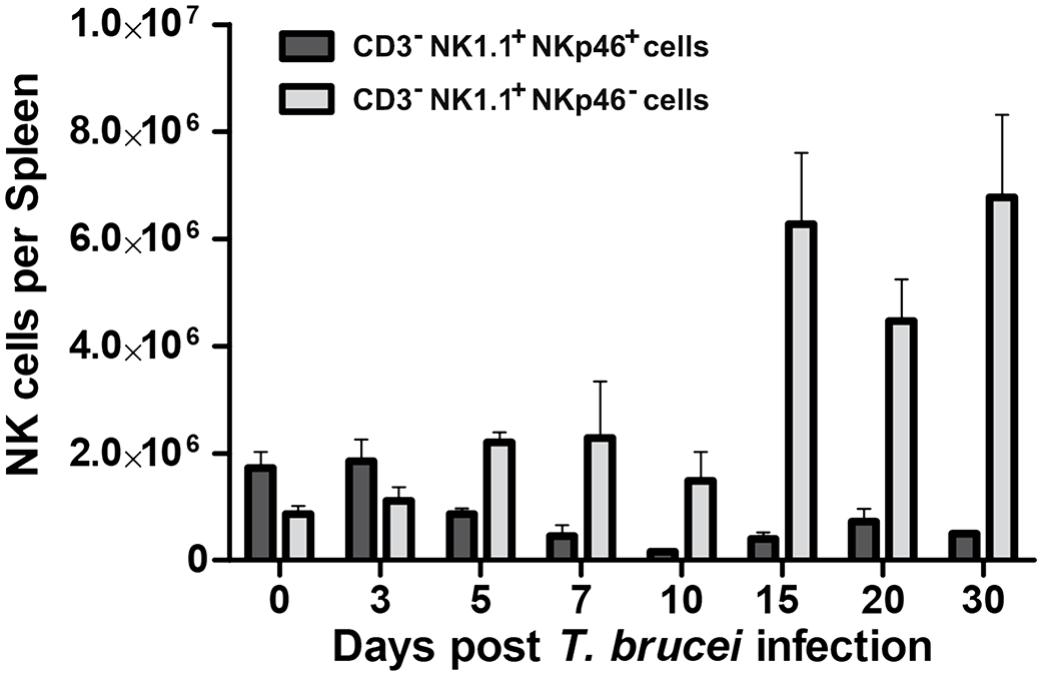
NKp46^+^ and NKp46^-^ NK cells in C57BL/6 mice infected with *T*. *brucei* AnTat 1.1 for up to 30 days. C57BL/6 mice (n = 3/group) were infected by ip inoculation with 5x10^3^ *T*. *brucei* AnTat1.1 or sham-infected by ip inoculation of physiologic saline. On days 0, 3, 5, 7, 10, 15, 20, and 30 after infection groups of infected and uninfected mice were killed, spleen cell suspensions made and analyzed by multicolor flow cytometry to identify NKp46^+^ and NKp46^-^ NK cells (CD3^-^NK1.1^+^) using FACs gates shown in S6 Fig. Data are presented as mean +/- 1 standard deviation; dark bars NKp46^+^ light bars NKp46^-^.

NKp46^-^ NK cells accumulated in the spleens of infected mice throughout 30 days of infection (Fig 10; representative FACs plots S6A–S6H Fig) and showed characteristic loss of CD49b as well as NKp46 expression, and gain of CD107a, FasL and TRAIL expression (S7 Fig, representative day 20 infected mouse). It is noteworthy that a small population of NKp46^-^ CD49b^-^ CD107a^+^ NK cells is also present in the spleens of naïve mice (S8 Fig). Thus the NKp46^-^ NK cells that accumulate in the spleens of *T*. *brucei* infected mice might derive from these cells. To determine whether that is the case, purified splenic NK cells (>99% NKp46^+^) were labeled with efluor 670 and 5x10^6^ injected iv into uninfected C57BL/6 mice or C57BL/6 mice that had been infected 7 days earlier with *T*. *brucei* AnTat 1.1. Recipient splenic efluor 670^+^ NK cells were analyzed 48 hours later for expression of NKp46 and CD107a (Fig 9). Similar numbers (10% of input) of efluor 670^+^ NK cells were recovered from the spleens of uninfected and infected recipients. Those in uninfected recipients retained expression of NKp46^+^ and did not acquire expression of CD107a^-^, whereas those in infected recipients had decreased levels of NKp46 and increased expression of CD107a, both of which were similar in level of expression to endogenous splenic NK cells in the infected mice (S9 Fig). These data suggest that NKp46^-^ CD49b^-^ CD107a^+^ NK cells derive from NKp46^+^ CD49b^+^ CD107a^-^ NK cells.

#### Transcriptome analysis

Analysis of differentiation antigen expression (Fig 7) and cytotoxic activity (Fig 9) of NK cells of naïve and *T*. *brucei* infected mice showed that the latter had greatly decreased levels of NK cell receptors and minimal killing capacity consistent with reprogramming and functional exhaustion. Dramatic differences in gene expression by purified day 10 post-infection and naïve NK cells were also observed by comparative RNA-seq (S10 Fig). Importantly, several of the transcribed genes that were decreased by more than log_2_ 1.5 in expression by NK cells in the day 10 infected mice compared to those of uninfected mice (Table 4) are known to be critical to NK cell development and function, namely genes encoding: i) transcription factors (*EOMES* and *TBX21* [T-bet]) which are required for NK cell development [54], ii) cytotoxic granule proteins (*Prf1* and *GZMA*) which are required for cell-mediated cytotoxicity; iii) NK cell receptors, (*KLRA18* [ly49], *NCR1* [NKp46], *KLRC1* [NKG2a], *KLRK1* [NKG2D] which regulate NK cell activation; iv) NK cell derived cytokines and chemokines (*LTB* and *CCL5*); and v) receptors for cytokines/chemokines and associated molecules that modulate NK cell proliferation (*IL-7Rα*, *IL-2Rβ/IL-15Rβ*, *IL-18R1*, *IL-18RAP*, *CXCR3*). In addition, among genes whose transcription was increased by more than log_2_ 1.5 in expression in the day 10 post infection NK cells compared to NK cells of uninfected mice was that for *IL-18bp* (IL-18 binding protein), which prevents IL-18 engaging the IL-18R on NK cells.

**Table 4:** Differential expression of selected genes in NK cells purified by FACS from pooled spleens (n = 5) of mice infected 10 days earlier with 5 x 10^3^ *T*. *brucei* ANTat 1.1E, or sham infected by administration of PBS.

Expressed gene	Log_2_ fold change	Mean counts
*EOMES* (T box family transcription family member)	-1.77	17,400
*TBX21* (T-bet; T box family transcription factor member)	-1.84	13,400
*Prf1* (perforin)	-1.48	4,880
*GZMA* (granzyme A)	-1.58	221,000
*GZMB* (granzyme B)	-0.0259	23,300
*NKG7* (NK granule protein 7)	-1.7	65,200
*FasL*	-1.6	2,980
*KLRA18* (Ly49)	-1.92	2,690
*NCR1* (NKp46)	-1.8	27,500
*KLRC1* (NKG2A)	-1.67	6,770
*KLRK1* (NKG2D)	-1.71	29,900
*CCL5* (rantes)	-1.64	124,000
*LTB* (TNF beta)	-1.63	6,040
*IL-7Rα*	-1.87	2,840
*IL-2Rβ/IL15Rβ*	-1.71	83,600
*CXCR3*	-1.7	5,740
*IL-18 R1*	-1.9	7,120
*IL-18Rap* (Il-18 receptor associated Protein)	-1.7	6,950
*IL-18bp* (IL-18 binding protein)	1.93	1920
*TCRαβγδ* *	Not expressed	
*C3Dγ* *	Not expressed	
*IgH* *	Not expressed	
*ITGAM* (Mac-1)*	Not expressed	

* The absence of genes encoding T cell, B cell and Macrophage markers in the RNA-seq database indicates the purity of isolated NK cells

## Discussion

Severe depletion of splenic B2 B cells within 10 days after infection with *T*. *b*. *brucei* Antat 1.1 is a property of infected C57BL/6 mice as well as other mouse strains. Several strains of mutant C57BL/6 mice (*TCR* ^*-/-*^, *C3* ^*-/-*^, *CD16* ^*-/-*^, *MyD88* ^*-/-*^, *TRIF* ^*-/-*^, *INFγ* ^*-/-*^, *TNFR1* ^*-/-*^, *Gal3* ^*-/-*^, *Prf1* ^*-/-*^) with defects in innate and adaptive immunity were examined for expression of this infection-induced, immunosuppressive trait. Only the gene encoding perforin (*Prf1)* had a non-redundant function required for rapid depletion of the splenic B2 B cells and loss of humoral immune function. Analyses over several weeks after infection showed that unlike *T*. *b*. *brucei* AnTat 1.1-infected C57BL/6 mice, infected *Prf1* ^*-/-*^ mice retained B cell follicles, developed high-titer IgM and IgG, particularly IgG1, antibody responses against a wide range of trypanosome polypeptides, controlled trypanosome parasitemia, maintained their body weight and blood packed cell volume, and survived at least twice as long as similarly infected intact mice, which jointly are characteristics of trypanosomiasis resistance [7, 15, 29, 55, 56]. Despite the dramatic decrease in trypanosomiasis pathology in infected *Prf1* ^*-/-*^ mice compared to infected intact mice, perforin gene deletion is not proposed as a practical approach to generate trypanosomiasis-resistant mammals. The mutation leaves cytotoxic T cells, NKT cells and NK cells reliant solely on death ligand-dependent killing of target cells and consequently would decrease their cytotoxic arsenal and increase host vulnerability to infections with intracellular pathogens. In our view, elucidation of the mechanism of *T*. *brucei* infection-induced, perforin-dependent, B cell killing is required before considering strategies to prevent this without compromising cell-mediated immunity.

Four complementary lines of evidence suggest that NK cells are solely responsible for the perforin-dependent depletion of splenic B cells in intact *T*. *brucei-*infected mice. Firstly, transitional and mature B2 B cell populations were depleted from the spleens of intact mice and mice lacking genes encoding both the beta and delta chains of the T cell antigen specific receptor and thus lacking all T cells and NKT cells, but were not depleted from infected intact and *TCR* ^*-/-*^ mice from which NK cells were removed by repeated administration of the NK1.1 specific IgG2a mAb PK136. In contrast the B2 B cells were depleted from infected mice that were administered an irrelevant myeloma IgG2a (done with intact C57BL/6 only). Secondly, splenic, liver and lymph node NK cells, but not T cells or NKT cells showed greatly increased surface expression of the cytotoxic granule degranulation marker CD107a during the period of B cell depletion consistent with selective execution of their cytolytic function. Thirdly, NK cells from uninfected and to a much lesser extent *T*. *brucei-*infected C57BL/6 mice killed splenic B cells during co-culture *in vitro*. Importantly, NK cells from uninfected or infected C57BL/6 mice did not kill B2 B cells from uninfected mice *in vitro* but did kill B2 B cells from infected mice indicating acquisition by these B cells of an NK cell activating phenotype. Fourthly, naïve NK cells that were transferred from intact C57BL/6 mice into *T*. *brucei*–infected *Prf1* ^*-/-*^ mice prevented infection-induced expansion of splenic B2 B cells consistent with *in vivo* B cell depletion.

NK cells typically kill virus-infected or transformed cells but do not kill healthy lymphocytes or other self-cells [57]. However, there are cases where this rule seems to be relaxed. Thus IL-2 activated human NK cells have been shown to kill stromal cells and antigen presenting cells *in vitro* [58]. In addition, NK cells have been shown to deplete virus-specific CD4^+^ T cells in mice with acute systemic LCMV infection with resultant suppression of adaptive immunity [59, 60]. They have also been shown to exacerbate pathology in mice exposed to high dose influenza virus infection although the mechanism is not resolved [61]. In addition, NK cells have been shown to regulate auto- and allo-reactive lymphocytes [62, 63] by pathways that include depletion of activated T cells [64, 65] and in which perforin-dependent cytolysis is implicated [66, 67]. Furthermore, several publications show that NK cells inhibit the development of antibody responses [68–70] and can inhibit the generation of virus-specific antibody and long-lived T- and B memory cells in a perforin-dependent manner [71]. These studies are consistent with a role for NK cells in down-regulation of adaptive immune responses per se, and with their more profound role in depletion of splenic B2 B cell and generalized immunosuppression in *T*. *brucei*-infected mice documented here.

Splenic NK cells from uninfected mice had a substantially higher capacity to kill *T*. *brucei* infection-modified B cells *in vitro* compared to NK cells isolated from the spleens of day 10 infected C57BL/6 mice. A similar *T*. *brucei* infection-induced loss of NK cell killing activity has been reported for tumor cell targets [72]. The decreased cytotoxic activity of the day 10 post-infection NK cells may result from their functional exhaustion following expression of cytotoxic activity during B cell depletion, evidenced by their greatly increased plasma membrane expression of the cytotoxic granule degranulation marker CD107a. In addition, NK cells that accumulated in the spleens of *T*. *brucei* infected mice after parasite wave remission had decreased expression of NKp46, CD49b and Ly49 receptors, an inability to populate the spleen of adoptive recipients, and decreased expression of genes that regulate NK cell development and function relative to mature splenic NK cells of uninfected mice including *EOMES*, *TBX21*, *Prf1*, *GZMA*, *KLRA18*, *NCR1*, *KLRC1*, *KLRK1*, *IL-5Rβ*, *IL-18R1*, *IL-18Rap*. Overall the data suggest, that the NKp46^-^CD107a^+^ NK cells that accumulate in the spleens of *T*. *brucei* infected mice have exhausted their cytotoxic potential, undergone partial reprogramming and will die rather than revert to normal mature splenic NK cells. If so, newly arising precursor cells would be required to sustain B2 B cell deletion throughout infection. In this regard adoptive transfer studies of efluor 670-labelled splenic NKp46^+^NK cells into naïve and *T*. *brucei* infected recipients indicated they were the precursors of the putative “spent NK cells” elicited by *T*. *brucei* infection, and further studies showed that NKp46^+^CD107a^-^ NK cells are sustained in the spleen throughout infection and hence may be responsible for on-going deletion of newly arising B2 B cells.

NKp46 is an NK cell activating receptor that is important in tumor killing [73–75]. It has been reported that aberrant CD56^Dim^ human NK cells with decreased surface expression of NKp46, NKp30, NKG2D and decreased cytotoxic function relative to CD56^Dim^ NK cells of uninfected newborns arise in the cord blood of newborns congenitally infected with *T*. *cruzi* [76]. Thus decreased expression of NKp46 by NK cells might be an outcome of exposure to infection-induced cytokine environments and consequently not limited to the *T*. *brucei* infected splenic environment. NKp46^-^ NK cells have also been shown to arise in the lungs of mice infected with a high dose of influenza virus and to exacerbate pathology leading to weight loss and death [61]. In addition, a loss of function mutation in the gene encoding NKp46 has been shown to result in NKp46^-^ NK cells that are hyper-responsive to various stimuli [77]. While it is possible that loss of NKp46 facilitates lymphocyte-destructive NK cell activity in influenza virus infected mice, that is unlikely to be the case in *T*. *brucei* infected mice because NK cells from naïve mice, which for the most part express NKp46, killed B cells from infected mice *in vitro* and had much higher cytotoxic activity than NKp46^-^ CD107a^+^ NK cells from the infected mice consistent with loss of cytolytic function of NK cells that had already degranulated.

NK cells integrate receptor and ligand interactions that are either activating or inhibitory [36]. Activated NK cells release cytotoxic granules at an immunological synapse [78] which forms between the NK cell and target cell after an activating threshold is achieved by an increase in activating ligands relative to inhibitory ligands on the target cell [79, 80]. MHC class I is a major inhibitory ligand for NK cells [36, 81], which consequently kill virus-infected and tumor cells that have decreased levels of MHC class I on their plasma membrane. In the case of *T*. *brucei*-infected mice, MHC class I expression is substantially increased, not decreased, on IgM^+^ cells (S11 Fig). However this does not automatically exclude loss of negative signaling through MHC class I receptors as a cause of NK cell-mediated B2 B cell destruction because splenic NK cells from day 10 *T*. *brucei* infected mice had decreased gene and protein expression of the Ly49 family of MHC class I receptors. Despite this, there is no compelling reason to consider that decreased expression of Ly49 receptors on NK cells is a requirement for *T*. *brucei* infection-induced B cell depletion. Thus NK cells from uninfected C57BL/6 mice, the majority of which express moderate or high levels of the Ly49 family of receptors, selectively killed B cells from *T*. *brucei* infected mice *in vitro*. Instead, we propose that the splenic B2 B cells from *T*. *brucei-*infected mice acquire potent NK cell activating ligand(s), which co-opt(s) NK cells to kill them. Many NK cell activating and co-stimulatory ligands have been reported [82] providing insights into possible infection-induced changes that would result in an NK cell activating phenotype. The identity of the ligand on post-infection B cells that tags the cells for NK cell mediated cytotoxicity is being addressed in on-going studies.

The studies presented above show that NK cells kill splenic B2 B cells in *T*. *brucei* infected mice by cell-mediated cytotoxicity and thus have a central role in infection-induced loss of humoral immune competence. Furthermore, the studies show that perforin-dependent cytotoxicity, possibly NK cell-mediated cytotoxicity, is required for development of anemia, weight loss and early mortality consistent with greatly increased NK cell degranulation in the spleen, liver and lymph nodes of the infected mice shown here. Further investigation is needed to identify the NK cell receptor(s) and B cell associated ligand(s) that mediate trypanosome infection-induced B cell killing, to determine how NK cell-mediated cytotoxicity interacts with other processes that contribute to trypanosomiasis-induced pathology [83–87], and importantly, to determine the contribution, if any, of NK cells to trypanosomiasis-pathology in host species other than mice, and in pathology associated with diseases in addition to African trypanosomiasis.

## Materials and Methods

### Ethics statement

The study was carried out in strict accordance with the recommendations in the Guide for the Care and Use of Laboratory Animals of the National Institutes of Health and Guidelines for the Use of Laboratory Animals in Research, Teaching and Testing of the International Council for Laboratory Animal Science. All animal studies were approved by the Institutional Animal Care and Use Committee, University of Massachusetts, Amherst, MA01003 USA, as documented in protocol #s 2010–0028, 2013–0049 and 2013–0050.

### Mice and trypanosomes

Male C57BL/6 (Taconic, Germantown, NY), B6-*129S4-C3<tm1Crr>/j* (*C3* ^*-/-*^), B6-*129P2-Fcgr3* ^*tm1Sjv*^ */J* (*CD16* ^*-/-*^), B6-*Cg-Lgals3* ^*tm1/poi*^ */J* (*Gal3* ^*-/-*^), B6-*prf1* ^*tm1sdz*^ */1* (*Prf1* ^*-/-*^), B6-*129P2-Tcrb* ^*tm1mom*^ *Tcrd* ^*tm1mom*^ */J* (*TCR* ^*-/-*^), B6-*Tnfrs1a* ^*tm1/mx*^ */J* (*TNF R1* ^*-/-*^), B6.129S7-*Ifng* ^*tm1ts*^/J (*INFγ* ^*-/-*^) mice were purchased from Jackson Laboratory (Bar Harbor, ME). All mice were housed under barrier conditions for at least 1 week after arrival at the university and were used at 7–9 week of age. Breeding pairs of B6-*MyD88* ^*-/-*^ and B6-*TRIF* ^*-/-*^ mice were a gift from Dr. Fitzgerald, University of Massachusetts School of Medicine (Worcester MA), were bred in the animal facilities at UMass Amherst and were used at 7 to 9 weeks of age. Mice were infected by intraperitoneal (i.p.) injection of 5000 exponentially growing pleomorphic *Trypanosoma brucei* Antat 1.1 or sham infected by i.p., injection of Dulbecco’s Phosphate Buffered Saline (PBS; GIBCO,Life Technologies); these parasites were derived from EATRO 1125 stock [88] and grown from cryopreserved parasites in immunocompromised (600r from a 127 Cesium source) C57BL/6 donors prior to injection. Parasitemia was assessed in tail blood by dilution in DPBS and counting using a hemocytometer.

### Mouse weight and packed cell volume measurements

Weight was measured using a Ohau brand digital scale. Blood packed cell volume was calculated after spinning blood in heparinized capillary tubes for 3 minutes in a hematocrit centrifuge (ADAMS MHCTII; BD, San Diego).

### Antibodies: Flow cytometry

Avidin-APC-Cy7 (San Diego), anti-CD49b-biotin (clone MD5-1), anti-FasL-PE (clone MFL3), anti-H2kb-PE (clone eBM2a), anti-NKp46-PE (clone 29A1.4), anti-IgM-PE (clone II/41), anti-NK1.1-APC (clone pk136), anti-Ly49 C/I/F/H-PE (clone 14B11), anti-Ly49-FITC (clone 3D10), anti-TRAIL-biotin (clone N2B2), anti-CD11b-APC-Cy7 (clone M1/70), anti-CD23-APC-Cy7 (clone B3B4), anti-CD23-FITC (clone B3B4), anti-CD45R (B220)-FITC (clone RA3-6B2), anti-CD93–APC (clone AA4.1), anti-CD107a (clone 1D4B) and anti-CD122-PE (Clone 5H4) were purchased from eBioscience (San Diego, CA). Anti-CD5-APC (clone 53–7.3), ant-CD11c-PE (clone HL3), anti-CD21-APC (clone 7G6), and anti-CD49b FITC were purchased from BD Biosciences (San Diego, CA). 7-amino-actinomycin D (7AAD) was purchased from EMD Chemicals (San Diego, CA). *IFA—*Anti-MOMA-1(biotin) AbCam was purchased from (Cambridge MA), avidin-FITC was purchased from BD Pharmingen (San Diego, Ca) and eFluor 615 anti-human/mouse CD45 (B220) was purchased from eBiosciences (SanDiego, Ca).

### Flow cytometric analysis

Mice were killed by lethal CO_2_ inhalation and their spleens were excised and mechanically dissociated in cold FACS Buffer (1.0% fetal bovine serum, FBS, [Atlanta Biologicals] in DPBS). Cell pellets were prepared by centrifugation (500 g, 10 min, 4°C) and suspended in 10ml of cold ammonium chloride red blood cell lysis buffer (ACK; 0.15M NH_4_Cl, 1.0 mM KHCO_3_, 0.1mM Na_2_-EDTA) and incubated for 4 minutes on ice. Remaining leukocytes were pelleted as above, washed twice in DPBS and suspended in FACs buffer. Aliquots (100 μl) containing 10^6^ cells were added to the wells of a 96 well V bottom plate, stained with combinations of specific monoclonal antibodies against B cell, T cell and NK cell differentiation antigens (listed in Table 1) and analyzed by multicolor flow cytometry as described by us [22]. Briefly, cells were incubated with Fc block (anti-CD16/CD32 Fc III/II, eBioscience, San Diego, CA; 1:1000 dilution) for 20 minutes at 4°C, pelleted, resuspended in 100 μl aliquots of biotin- or fluorochrome-conjugated primary antibodies (listed above) for 30 minutes at 4°C, washed twice in cold FACs buffer, resuspended in 100 μl aliquots of FACs buffer with or without streptavidin (SA) conjugated fluorochromes and incubated for an additional 30 minutes at 4°C. Samples were washed twice and resuspended in 300 μl FACs buffer with 1 μg of 7AAD, a fluorescent DNA intercalating agent that binds to DNA in membrane permeable (dead or dying) cells, (EMD Chemicals, San Diego, CA). Analyses were performed using a flow cytometer (LSRII BD Biosciences, San Jose, CA) and data processed using FLOWJO software (Tree Star Inc., Ashland, OR) to determine the percentages of 7AAD^-^ and where stated also 7AAD^+^ cells in antibody and fluorochrome-defined subsets. The total number of cells in each population was determined by multiplying the percentages of subsets within a series of marker negative or positive gates by the total cell number of viable leukocytes in the donor spleen.

### Immunofluorescence staining of the mouse spleen

Spleens from uninfected and infected C57BL/6 and *Prf1* ^*-/-*^ mice were embedded in OCT (Sakura Finetek, Torrance, CA), frozen in dry ice, stored frozen at -80°C and incubated at -20°C for 30 minutes before cryostat sectioning. Frozen sections (10) μm were cut and adhered to Poly-Prep slides (Sigma-Aldrich, St. Louis, MO), air dried and fixed in ice cold acetone for 10 minutes. Slides were blocked with 5% bovine serum albumin (BSA) and Fc blocker CD16/CD32 (eBioscience) for 30 minutes and stained with biotin-conjugated antibody MOMA-1 at room temperature for one hour. Sections were washed and treated for one hour at room temperature with Avidin FITC and antibody B220. Stained sections were washed, air dried at room temperature and mounted with ProLong Gold antifade reagent (Life Technologies) and covered with glass coverslips (Corning). All the slides were read on a Zeiss MOT200 inverted microscope with a Zeiss apotome at 20x magnification.

### NK cell depletion by mAb PK136 [41, 43]

Mice were injected intraperitoneally with 500 ug anti-NK1.1 IgG2a monoclonal antibody purified from hybridoma (PK136, ATCC Manassas, Virginia) culture supernatant by Protein G chromatography, or with an irrelevant control IgG2a immunoglobulin (M9144, Sigma-Aldrich). Treatments were repeated on days 4 and 7 after the first injection of anti-NK1.1 and splenic B cell, T cell and NK cell populations assessed on days 3, 7 and 10 using antibody staining and multicolor flow cytometry as described above.

### Isolation of splenic B cells and NK cells, and labeling with E-fluor670

Erythrocyte depleted spleen cell suspensions were prepared as discussed for flow cytometry. NK cells and B cells were isolated by negative selection using respectively Miltenyi kits (#130-096-892 and #130-095-831). The yield of NK cells was 1.5% to 2.0% of input spleen cells and >98% of these were CD3^-^NK1.1^+^ by staining and flow cytometry analysis (Becton Dickenson, Fortessa). The yield of splenic B cells was 15% to 18% of input spleen cells from both naïve mice and day 8 infected mice, and >98% of these cells were B220^+^IgM^+^ by staining and flow cytometry. For tracking studies these cells were labeled with eFluor*670 (eBiosciences Affimetrix #65-0840-85) which did not affect viability.

### NK cell cytotoxic assay

NK cell cytotoxicity assays were performed in 96 well V bottom plates at 37°C in a humid atmosphere of 5% CO_2_ in air. Each well contained 200 μl medium (RPMI 1640 supplemented with 10% Hyclone fetal bovine serum, Penicillin Streptomycin, L-glutamine and 0.2 μg rat anti-mouse anti-CD40 mAb 3/23/ml medium Becton and Dickenson) and 10^5^ eFluor 670-labelled B cells with or without NK cells to give ratios of between 50 NK cells/ 1B cell and 1 NK cell/4 B cells. Incubations were for 3 hours after which each well was inoculated with the same number of fluorescent beads (Accudrop, Becton and Dickenson) and 7AAD prior to flow cytometric analysis to allow determination of viable B cells (eFluor 670^+^ 7AAD^-^) relative to bead number and thus the number of viable B cell remaining in each culture. Results are presented as: relative viable B cell recovery (where 1 = number of viable B cells after incubation in the absence of NK cells).

### Western blot analysis of trypanosome-polypeptide specific antibodies in mouse serum

A pellet containing 10^8^ *T*. *brucei* AnTat 1.1 purified from the blood of infected irradiated mice by DEAE52 chromatography [89] and washed twice in PBS was suspended in 5 ml lysis buffer (PBS, containing 0.5%NP40 and protease inhibitor cocktail [Complete mini tablets, Roche, Indianapolis, IN]) and incubated at room temperature for 30 minutes. Insoluble material present in the lysate was removed by centrifugation at 1000g for 5 mins. Protein content was determined by Bradford assay and an aliquot (600ug protein content) of trypanosome lysate was fractionated by reducing SDS-PAGE on a 10% polyacrylamide gel in a single gel-spanning slot and separated polypeptides were transferred to a polyvinylidene difluoride membrane using a mini trans-blot transfer apparatus (Bio-Rad) according to the manufacturer’s protocols. The membrane was blocked with 5% nonfat milk in 10 mM Tris, pH 8.0, 150 mM NaCl, 0.5% Tween 20 (TBST) for 60 min, washed once with TBST, inserted in a slot blot apparatus (Mini Protean Multi Screen II, Biorad) and serum, prepared from blood of control and trypanosome-infected mice (1:200 dilution, 600 μl/slot), was added to individual slots and incubated at 4°C for 2 h. Membranes were washed three times (10ml TBST, room temperature, 10 min) and incubated with horseradish peroxidase-conjugated anti-mouse IgM, or IgG_1_ antibodies (ABCAM 0.6 μg antibody/ml TBST) for 1 hour, then washed 3 times with TBST before developing with chemiluminescence substrate (ThermoPierce).

### Enzyme linked immunosorbent assay (ELISA) of trypanosome-specific antibodies

Wells in a 96 well plate (NUNC) were coated with 0.1 μg *T*. *brucei* Antat1.1E lysate (prepared as above) in 100 μl PBS containing 0.05% NP40, washed with ELISA wash buffer (PBS + 1% bovine serum albumin [BSA] + 0.05% TWEEN20) and non-specific protein binding sites were blocked by incubation with 200 μl aliquots of PBS + 1% BSA. Aliquots (100 μl) of control and post-infection mouse serum diluted in PBS were added to each well, incubated for 2 hours at room temperature and wells were washed 3 times with ELISA wash buffer to remove unbound serum immunoglobulins. Bound antibodies were revealed by addition of biotinylated anti-mouse IgM or anti-mouse IgG (BD biosciences) to each well and bound second step antibody was quantified after washing by incubation with avidin-conjugated horse radish peroxidase (BD biosciences). Captured antibody was visualized with Ultra TMB ELISA (Thermo scientific) and absorbance readings were made at 450nm, using a 96 well plate spectrophotometer.

### mRNA seq

NK cells (>99.8% CD3^-^ NK1.1^+^) were isolated using a fluorescence activated cell separator from cell suspensions of pooled spleens of 5 naïve C57BL/6 mice and 5 C57BL/6 mice that had been infected 10 days earlier with 5 x10^3^ *T*. *brucei* Antat 1.1. Total DNAse treated RNA was extracted from purified NK cells using a RNeasyPlus Mini Kit (Qiagen), amount of isolated RNA estimated by on-chip electrophoresis using an Agilent RNA 6000 nano kit on an Agilent 2100 Bioanalyzer (Agilent Technologies), and its integrity confirmed by a RIN number of 9 or greater. mRNA libraries were prepared from isolated RNA using a TruSeq Stranded mRNA Library LT kit (Illumina) as recommended by manufacturer. Briefly, polyA containing mRNA was isolated from total RNA, fragmented, primed for cDNA synthesis, reverse transcribed to cDNA using Superscript II reverse transcriptase and second strand synthesis performed using dUTP instead of dTTP to quench 2^nd^ strand amplification during subsequent PCR. Double stranded (ds) cDNA was separated from the 2^nd^ strand mix using AMPure XP beads (Beckman Coulter), a single “A” nucleotide added at 3’ ends, the preparation end ligated with proprietary bar coded paired end adapters each bearing a single T nucleotide at 3’ ends and unique to each library, and tagged ds cDNA isolated using AMPure XP beads. cDNA fragments with adapter molecules at both ends were amplified using a primer cocktail that anneals to the ends of the adapters, the quality of the resulting library was estimated by on-chip electrophoresis (Agilent DNA 1000 chip) on Qubit 3.0 Fluorometer (ThermoFisher Scientific) and single run sequencing (2 x 150) was performed using a NextSeq 500 mid output kit on a NextSeq 500 sequencer (Illumina). The sequences were trimmed at the 3’ end to 125 bp using the FASTAQ Toolkit and analyzed using RNA express in BaseSpace (Illumina); sequence alignment analysis was performed against Mus musculus UCSC reference genes within the BaseSpace program.

### Statistics

Cell population and other data obtained from infected animals and from non-infected controls were subjected to two-tailed T tests with significant differences reported as follows: p≤0.05, (**) p≤0.01, (***) p≤0.001. Differences among multiple groups were analyzed using ANOVA and means were compared using Tukey’s honest significant difference (HSD) test when p≤0.05 (GraphPad Prism v.4.0, GraphPad Software Inc. San Diego, CA).

## Supporting Information

S1 Fig Transitional and splenic mature B2 B cell gating strategy. Representative plots obtained using spleen cells from uninfected mice stained B cell populations as described in table 1. **(A)** Spleen leukocytes cells gated by side scatter [SSC] and forward scatter [FSC], **(B)** viable splenocytes gated by lack of uptake of the vital dye 7AAD, **(C)** viable splenocytes gated by surface staining with H2D^b^ and IgM, **(D)** viable splenic B cells 7AAD^-^ H2-D^b+^ IgM^+^ gated as follicular B cell [FoB] and marginal zone B cells [MZB] based on expression levels of CD23 and CD21, **(E)** viable transitional B cells gated as 7AAD^-^ B220^+^ AA4.1^+^, **(F)** viable Transitional Type 1 B cells gated by 7AAD^-^ B220^+^ CD23^-^ IgM^+^, **(G)** viable Transitional Type 2 and 3 B cells gated by 7AAD^-^ B220^+^ CD23^+^ and respectively IgM^lo^ and IgM^hi^.
(TIF)

S2 Fig B2 B cell depletion in *Infγ* ^*-/-*^ mice. **(A-C)** Transitional B Cells (T2+T3; A), marginal zone B cells (MZB; B) and follicular B cells (FoB; C) in spleens of uninfected (day 0) and *T*. *brucei* AnTat 1.1 infected (day 10) *Infγ* ^*-/-*^ C57BL/6 mice (n = 3). Significance (<0.01m in all panels) was determined using one-way ANOVA and Tukey’s HSD test comparing uninfected controls to infected individuals. Results are representative of 2 identical experiments. FACS plots of transitional B cells from a representative uninfected (D) and infected (E) mouse, and of FoB and MZB cells from the same uninfected (F) and infected (G) mice.
(TIF)

S3 Fig Impact of NK1.1 cell depletion on splenic B2 B cell survival in *TCR* ^*-/-*^ C57BL/6 mice infected with *T*. *brucei* AnTat 1.1. *TCR* ^*-/-*^ C57BL/6 mice (n = 3/group) were administered 500ug mAb PK136 anti-NK1.1 monoclonal antibody ip on days 0, 3, and 7 after infection with *T*. *brucei* Antat 1.1 (day 10) or after sham infection (day 0). Spleen cells were stained for surface markers that define: **(A)** Transitional B cell, **(B)** Marginal Zone B cells, and **(c)** Follicular B cells as described in Table 1 and analyzed using flow cytometry. There was no significant difference between B cell numbers in the uninfected and infected mice, determined using one-way ANOVA and Tukey’s HSD test. Results are representative of 2 identical experiments.
(TIF)

S4 Fig B2 B cell depletion in CD16^-/-^ mice. **(A-C)** Transitional B Cells (T1, T2, T3), **(D)** marginal zone B cells (MZB) and **(E)** follicular B cells (FoB) in spleens of uninfected (day 0) and *T*. *brucei* AnTat 1.1 infected (day 10) *CD16* ^*-/-*^ C57BL/6 mice (n = 3). Significance (*<0.05) was determined using one-way ANOVA and Tukey’s HSD test comparing uninfected controls to infected individuals. Results are representative of 2 identical experiments.
(TIF)

S5 Fig CD107a expression by liver and lymph node NK cells of uninfected and *T*. *brucei* infected mice. Representative FACS plots of CD107a expression on NK cells (NK1.1^+^ CD3^-^) present in leukocyte cell suspensions prepared from liver (A) and lymph node (B) of a representative uninfected mouse (blue line) and a mouse that had been infected 10 days earlier with *T*. *brucei* AnTat 1.1 (red line).
(TIFF)

S6 Fig FACS plots of NKp46 expression by splenic NK cells in *T*. *brucei* AnTat 1.1-infected mice. The figure presents FACS plots of NKp46^+^ and NKp46^-^ NK cells [CD3- NK1.1+] in spleen cell suspensions of a representative: **(A)** uninfected mouse and mice that had been infected for **(B)** 3 days, **(C)** 5 days, **(D)** 7 days, **(E)** 10 days, **(F)** 15 days, **(G)** 20 days, **(H)** 30 days following ip inoculation of 5 x 10^3^ *T*. *brucei* AnTat 1.1.
(TIF)

S7 Fig Splenic NK cell differentiation antigen expression at 20 days after infection with *T*. *brucei* AnTat 1.1. Mice were infected with 5x10^3^ *T*. *brucei* AnTat 1.1 or sham-infected by ip inoculation of phosphate buffered saline and killed 20 days later. Spleen cells were stained with specific mAbs and analyzed by FACS to determine the expression of CD107a, CD49b, NKp46, FasL and TRAIL by NK cells (CD3- NK1.1+). Results from a representative infected (red lines) and uninfected (blue lines) mouse are shown.
(TIF)

S8 Fig Expression of CD107a by CD49b^-^NKp46^-^ and CD49b^+^NKp46^+^ splenic NK cells of a representative uninfected C57BL/6 mouse. (TIF)

S9 Fig Fate analysis of adoptively transferred NK cells. Splenic NK cells were purified from C57BL/6 mice, labeled with efluor 670 and injected iv into uninfected mice or mice that had been infected 7 days earlier with 5x10^3^ *T*. *brucei* AnTat 1.1. After 48 hours spleen cell suspensions were prepared and analyzed by multicolor flow cytometry for expression of NKp46 and CD107a by efluor 670^+^ (transferred) and efluor 670^-^ (resident) NK cells (CD3^-^NK1.1^+^). **(A,B)** solid line—resident NK cells, dotted line—transferred NK cells, **(C,D)** solid line—resident NK cells in an uninfected mouse, dashed line—resident NK cells in a day 9 infected mouse, dotted line—transferred NK cells in a day 9 infected mouse.
(TIF)

S10 Fig Summary of NK cell RNA-seq data. Transcriptome analysis of FACS purified NK cells (CD3^-^NK1.1^+^) from pooled spleens of uninfected (n = 5) and day 10 *T*. *brucei* AnTat 1.1 infected (n = 5) mice (2 x 150bp reads Illumina).
(TIF)

S11 Fig Expression of class I MHC on splenic IgM^+^ cells of uninfected and *T*. *brucei* Antat1.1-infected C57BL/6 mice. Splenocytes from uninfected C57BL/6 mice and mice infected 10 days earlier with *T*.*brucei* Antat1.1 were stained with monoclonal antibodies specific for IgM and MHCI and analyzed by flow cytometry. Blue lines represent cells from representative uninfected animals and red lines represent cells from representative infected animals. Results are representative of 3 identical experiments.
(TIF)
