# Supplementary figures and images for: Trypanosoma brucei Co-opts NK Cells to Kill Splenic B2 B Cells
Source: PLoS Pathog. 2016 Jul 12;12(7):e1005733. doi: 10.1371/journal.ppat.1005733 (PMC4942092; doi:10.1371/journal.ppat.1005733)

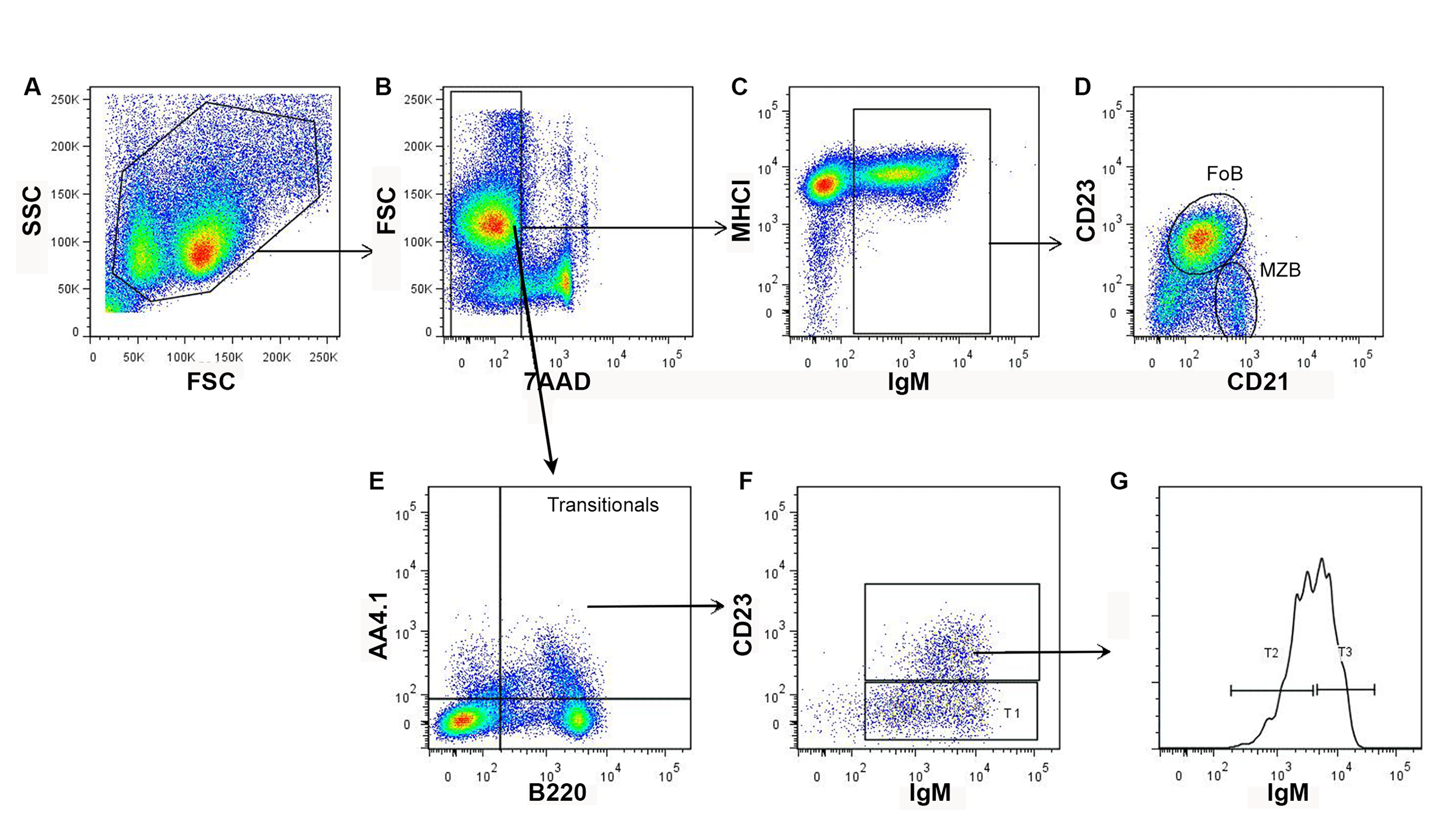

Supplement: S1 Fig — Representative plots obtained using spleen cells from uninfected mice stained B cell populations as described in table 1. (A) Spleen leukocytes cells gated by side scatter [SSC] and forward scatter [FSC], (B) viable splenocytes gated by lack of uptake of the vital dye 7AAD, (C) viable splenocytes gated by surface staining with H2Db and IgM, (D) viable splenic B cells 7AAD- H2-Db+ IgM+ gated as follicular B cell [FoB] and marginal zone B cells [MZB] based on expression levels of CD23 and CD21, (E) viable transitional B cells gated as 7AAD- B220+ AA4.1+, (F) viable Transitional Type 1 B cells gated by 7AAD- B220+ CD23- IgM+, (G) viable Transitional Type 2 and 3 B cells gated by 7AAD- B220+ CD23+ and respectively IgMlo and IgMhi. (TIF) [file ppat.1005733.s001.tif]

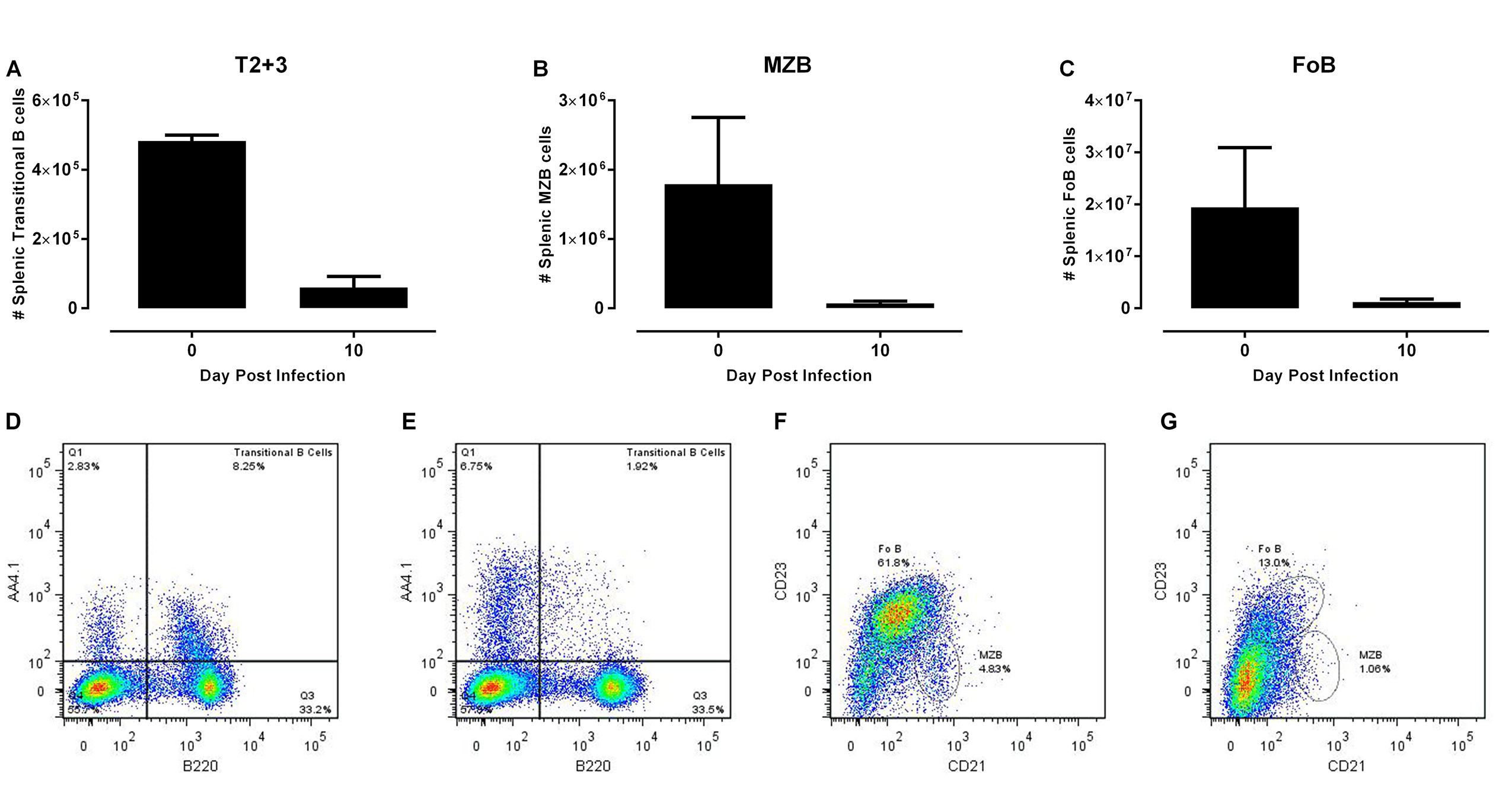

Supplement: S2 Fig — (A-C) Transitional B Cells (T2+T3; A), marginal zone B cells (MZB; B) and follicular B cells (FoB; C) in spleens of uninfected (day 0) and T. brucei AnTat 1.1 infected (day 10) Infγ -/- C57BL/6 mice (n = 3). Significance (<0.01m in all panels) was determined using one-way ANOVA and Tukey’s HSD test comparing uninfected controls to infected individuals. Results are representative of 2 identical experiments. FACS plots of transitional B cells from a representative uninfected (D) and infected (E) mouse, and of FoB and MZB cells from the same uninfected (F) and infected (G) mice. (TIF) [file ppat.1005733.s002.tif]

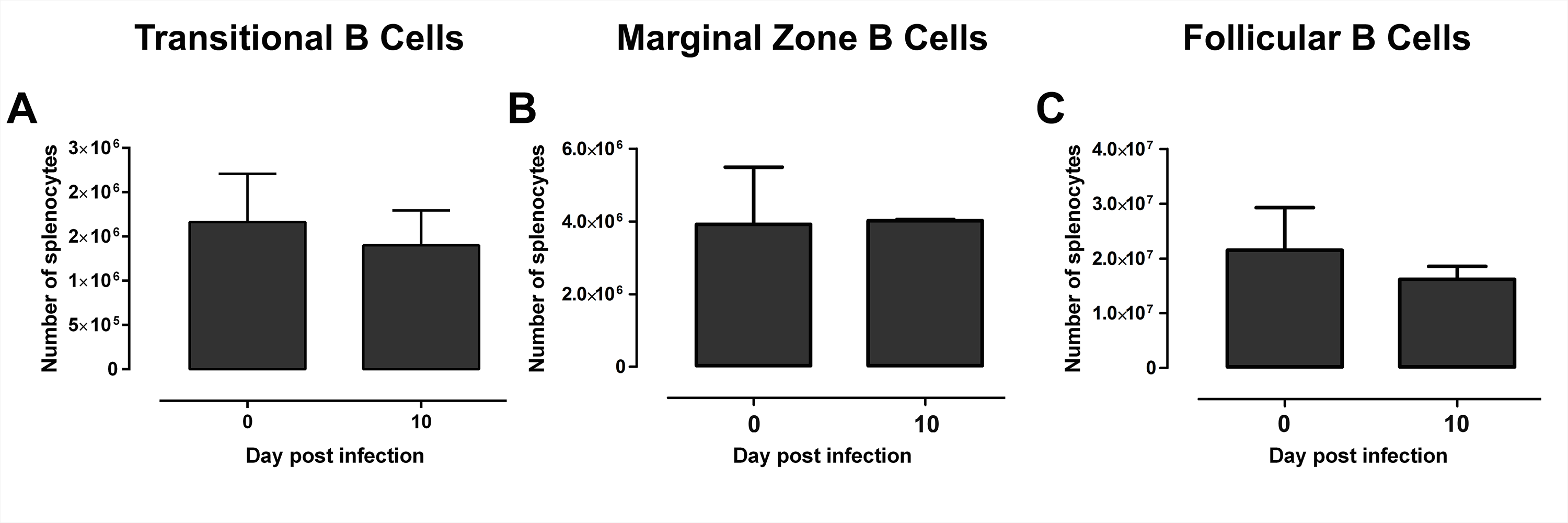

Supplement: S3 Fig — TCR -/- C57BL/6 mice (n = 3/group) were administered 500ug mAb PK136 anti-NK1.1 monoclonal antibody ip on days 0, 3, and 7 after infection with T. brucei Antat 1.1 (day 10) or after sham infection (day 0). Spleen cells were stained for surface markers that define: (A) Transitional B cell, (B) Marginal Zone B cells, and (c) Follicular B cells as described in Table 1 and analyzed using flow cytometry. There was no significant difference between B cell numbers in the uninfected and infected mice, determined using one-way ANOVA and Tukey’s HSD test. Results are representative of 2 identical experiments. (TIF) [file ppat.1005733.s003.tif]

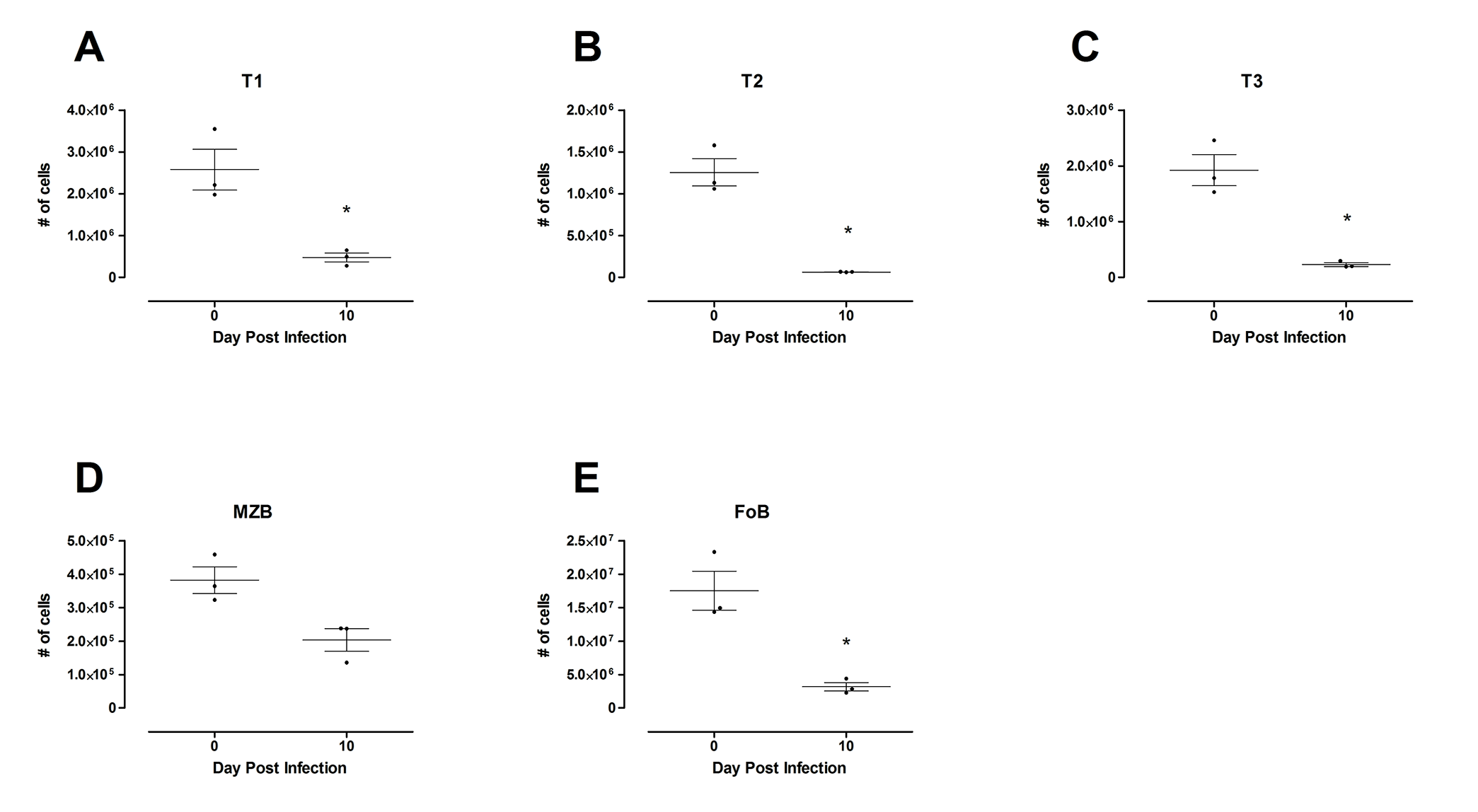

Supplement: S4 Fig — (A-C) Transitional B Cells (T1, T2, T3), (D) marginal zone B cells (MZB) and (E) follicular B cells (FoB) in spleens of uninfected (day 0) and T. brucei AnTat 1.1 infected (day 10) CD16 -/- C57BL/6 mice (n = 3). Significance (*<0.05) was determined using one-way ANOVA and Tukey’s HSD test comparing uninfected controls to infected individuals. Results are representative of 2 identical experiments. (TIF) [file ppat.1005733.s004.tif]

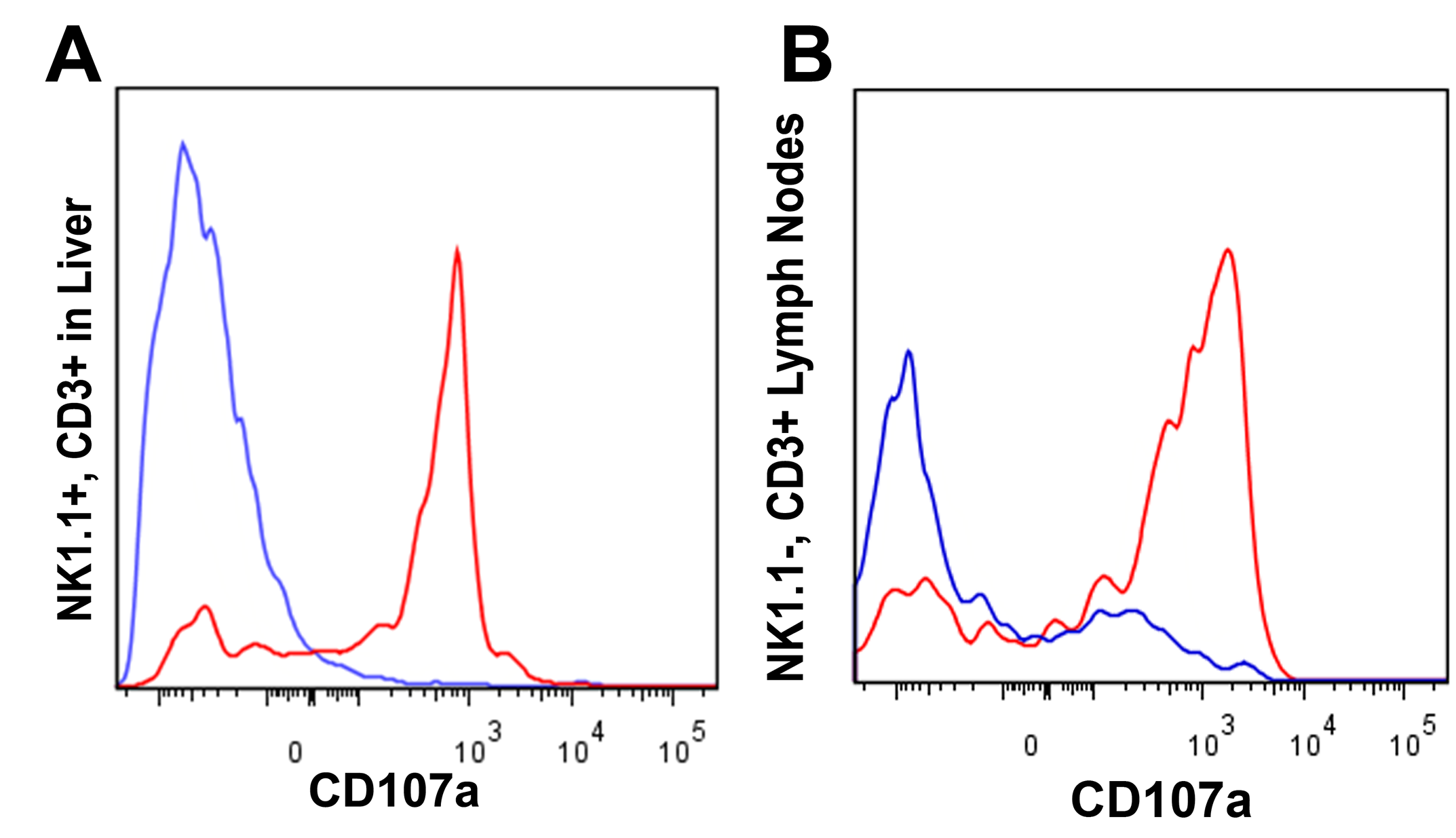

Supplement: S5 Fig — Representative FACS plots of CD107a expression on NK cells (NK1.1+ CD3-) present in leukocyte cell suspensions prepared from liver (A) and lymph node (B) of a representative uninfected mouse (blue line) and a mouse that had been infected 10 days earlier with T. brucei AnTat 1.1 (red line). (TIFF) [file ppat.1005733.s005.tiff]

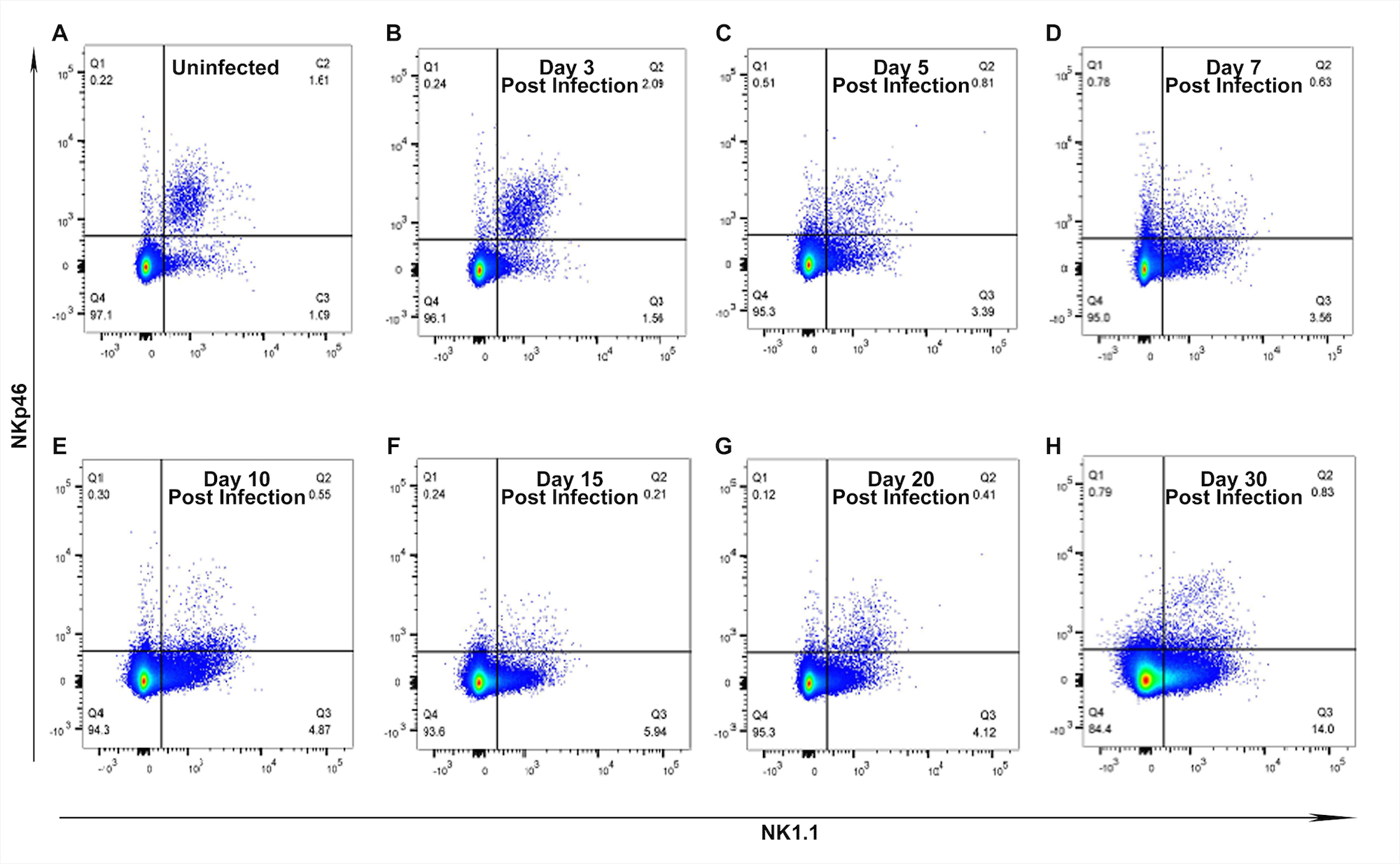

Supplement: S6 Fig — The figure presents FACS plots of NKp46+ and NKp46- NK cells [CD3- NK1.1+] in spleen cell suspensions of a representative: (A) uninfected mouse and mice that had been infected for (B) 3 days, (C) 5 days, (D) 7 days, (E) 10 days, (F) 15 days, (G) 20 days, (H) 30 days following ip inoculation of 5 x 10^3^ T. brucei AnTat 1.1. (TIF) [file ppat.1005733.s006.tif]

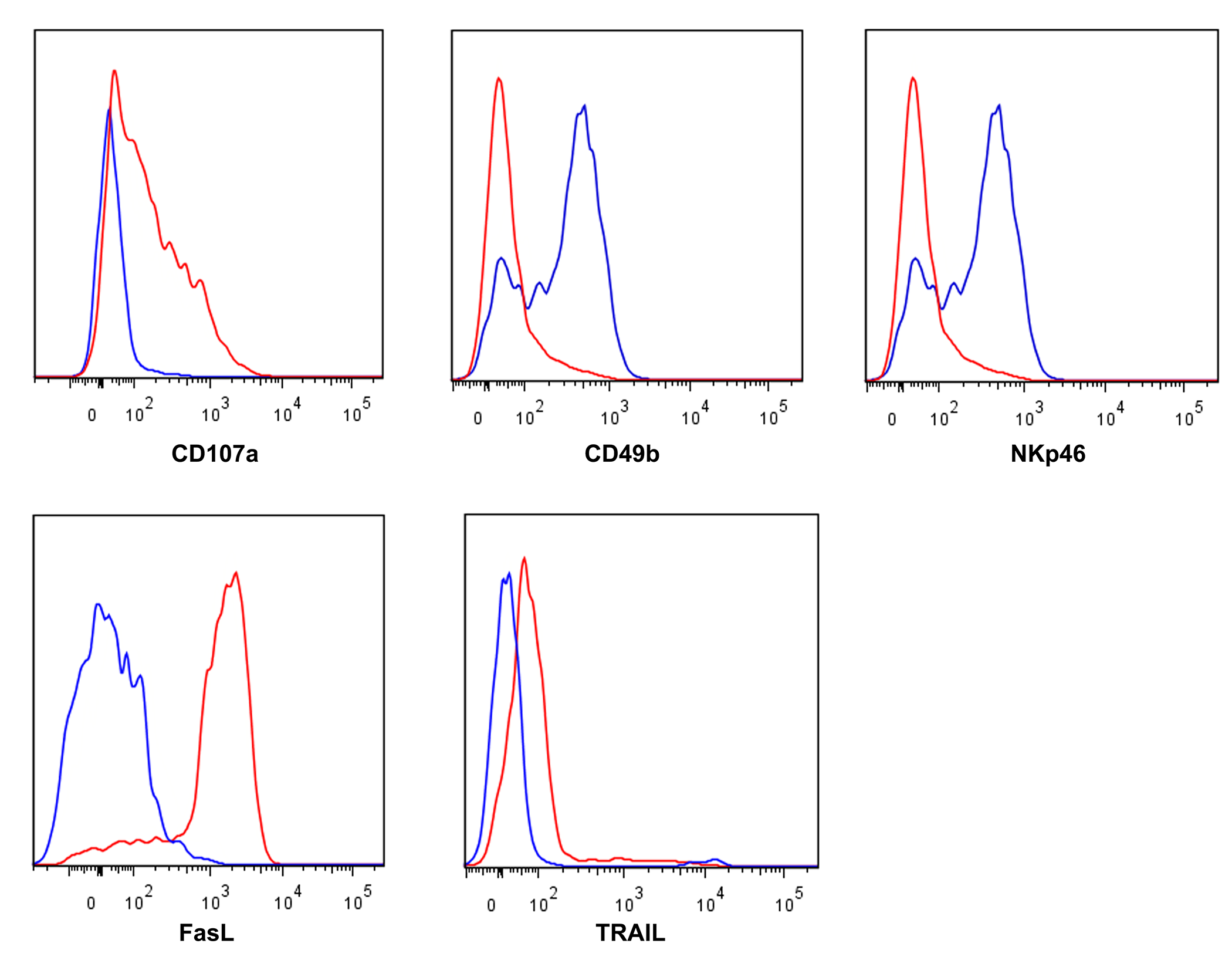

Supplement: S7 Fig — Mice were infected with 5x10^3^ T. brucei AnTat 1.1 or sham-infected by ip inoculation of phosphate buffered saline and killed 20 days later. Spleen cells were stained with specific mAbs and analyzed by FACS to determine the expression of CD107a, CD49b, NKp46, FasL and TRAIL by NK cells (CD3- NK1.1+). Results from a representative infected (red lines) and uninfected (blue lines) mouse are shown. (TIF) [file ppat.1005733.s007.tif]

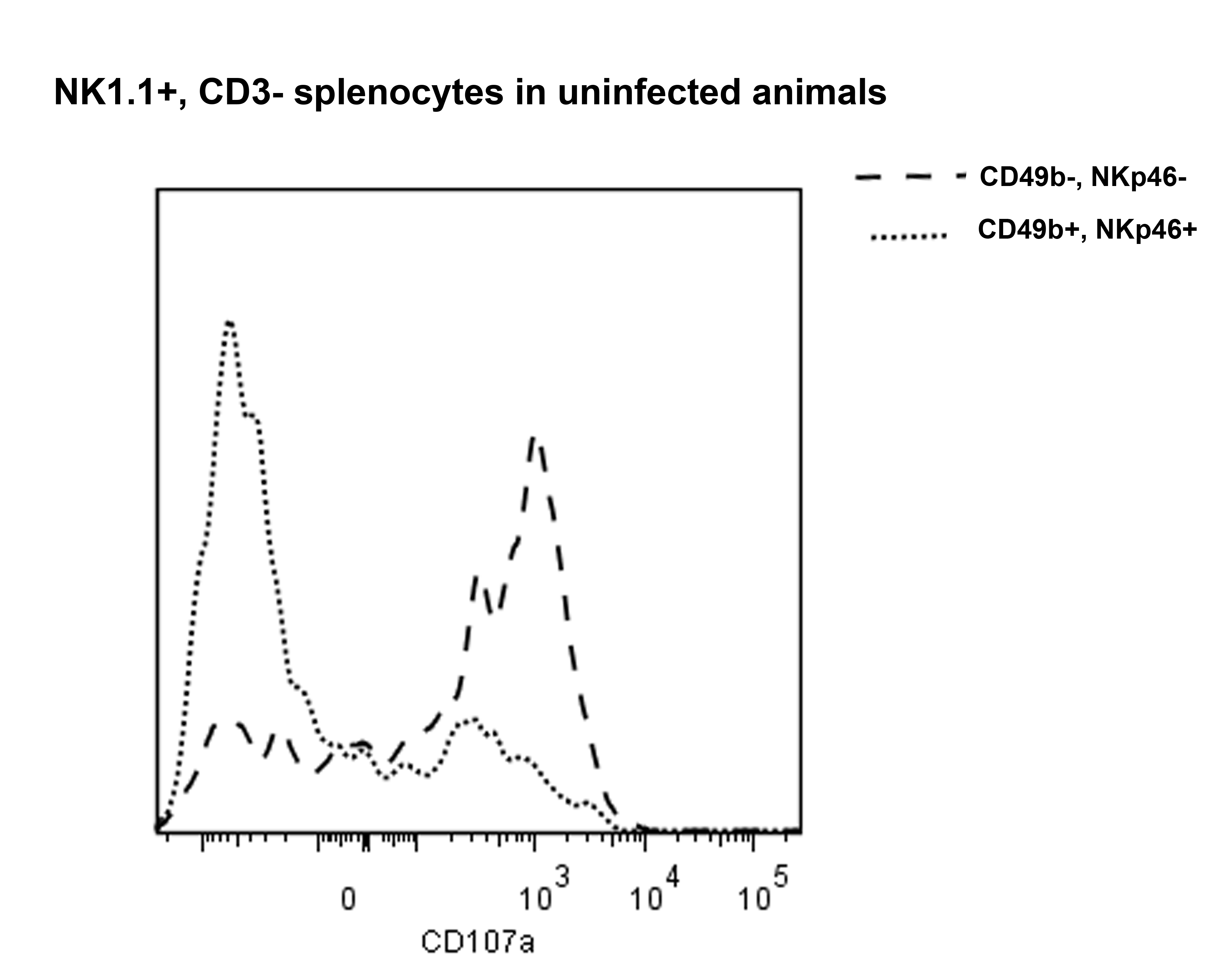

Supplement: S8 Fig — (TIF) [file ppat.1005733.s008.tif]

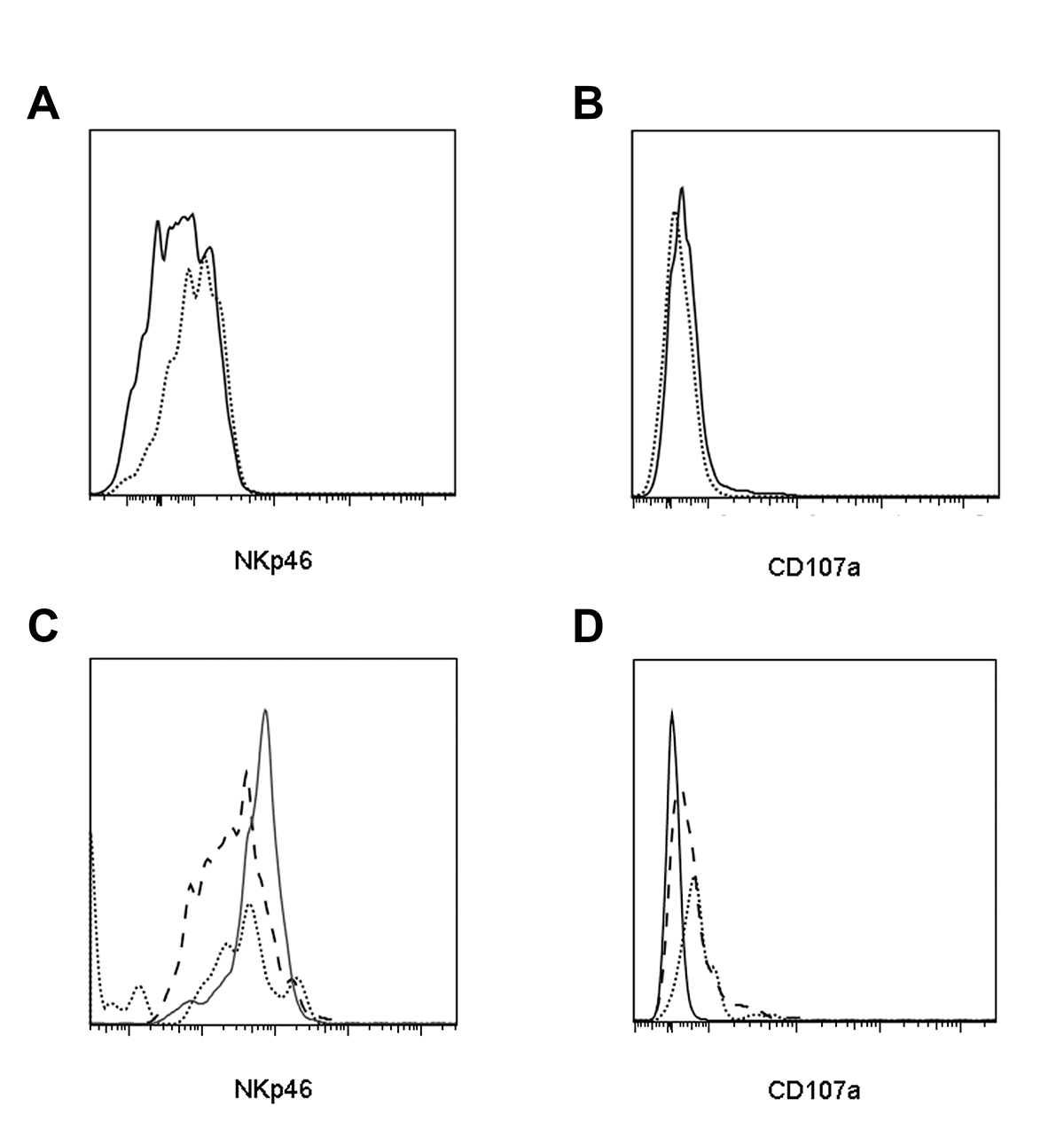

Supplement: S9 Fig — Splenic NK cells were purified from C57BL/6 mice, labeled with efluor 670 and injected iv into uninfected mice or mice that had been infected 7 days earlier with 5x10^3^ T. brucei AnTat 1.1. After 48 hours spleen cell suspensions were prepared and analyzed by multicolor flow cytometry for expression of NKp46 and CD107a by efluor 670+ (transferred) and efluor 670- (resident) NK cells (CD3-NK1.1+). (A,B) solid line—resident NK cells, dotted line—transferred NK cells, (C,D) solid line—resident NK cells in an uninfected mouse, dashed line—resident NK cells in a day 9 infected mouse, dotted line—transferred NK cells in a day 9 infected mouse. (TIF) [file ppat.1005733.s009.tif]

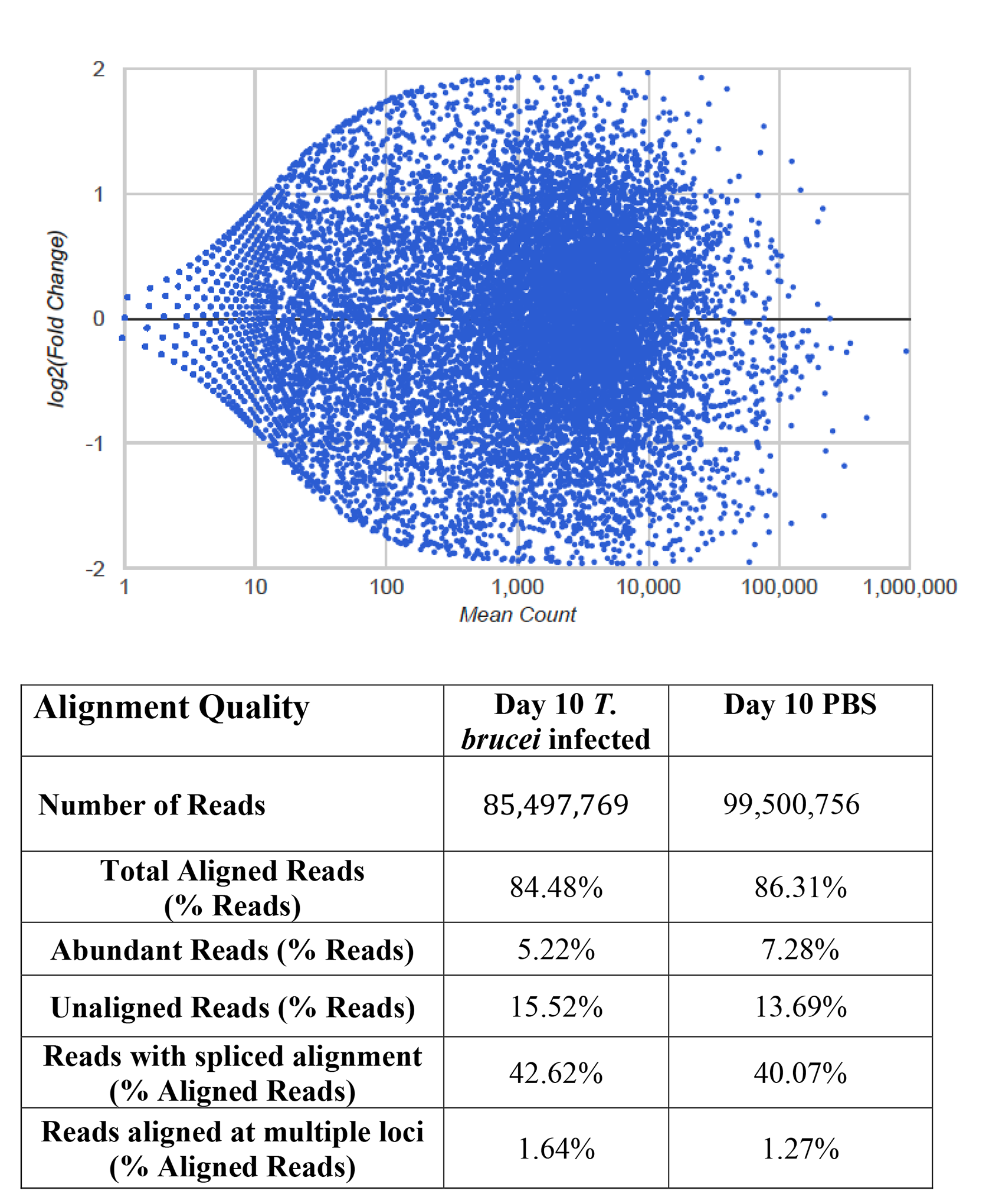

Supplement: S10 Fig — Transcriptome analysis of FACS purified NK cells (CD3-NK1.1+) from pooled spleens of uninfected (n = 5) and day 10 T. brucei AnTat 1.1 infected (n = 5) mice (2 x 150bp reads Illumina). (TIF) [file ppat.1005733.s010.tif]

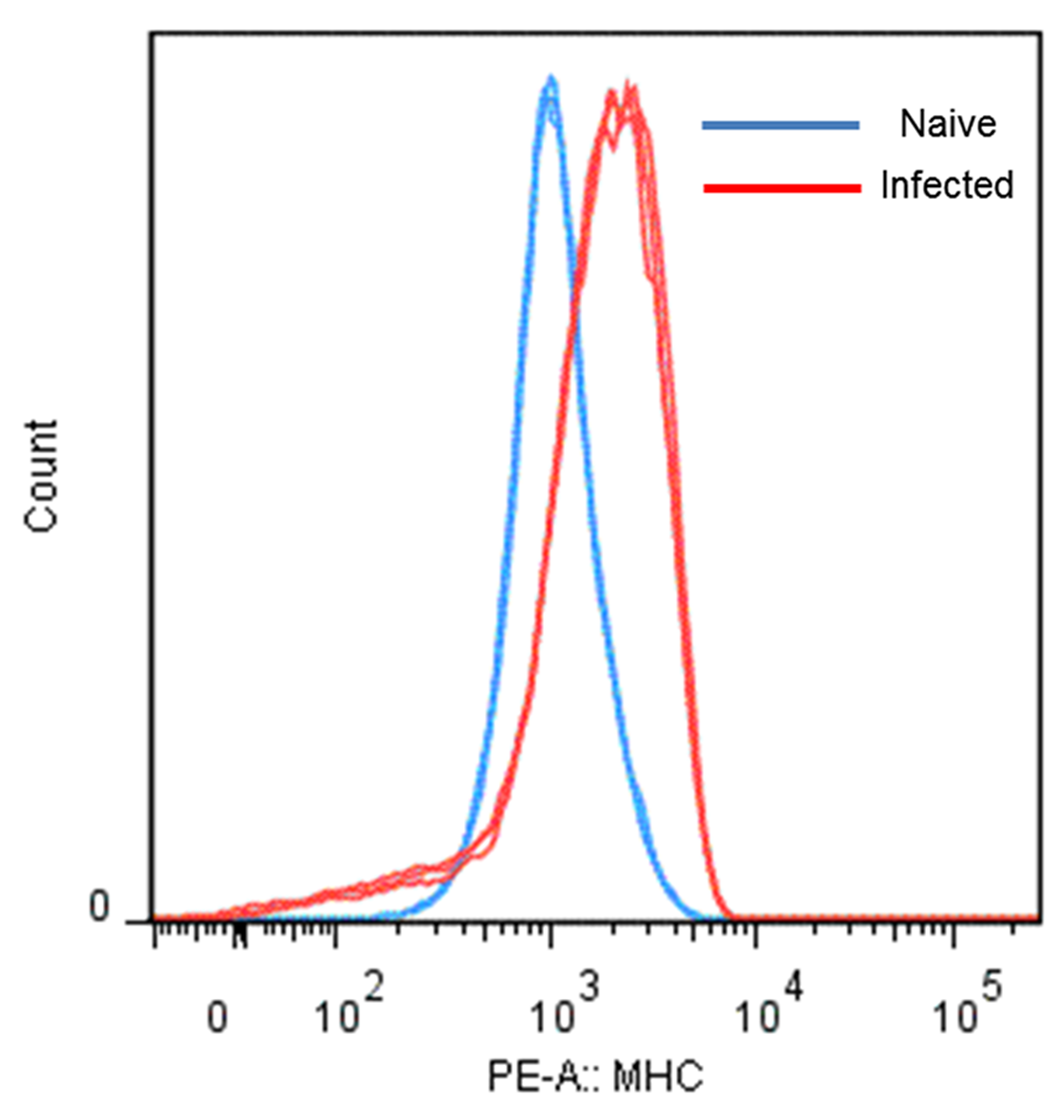

Supplement: S11 Fig — Splenocytes from uninfected C57BL/6 mice and mice infected 10 days earlier with T.brucei Antat1.1 were stained with monoclonal antibodies specific for IgM and MHCI and analyzed by flow cytometry. Blue lines represent cells from representative uninfected animals and red lines represent cells from representative infected animals. Results are representative of 3 identical experiments. (TIF) [file ppat.1005733.s011.tif]
